# EGFR-dependent aerotaxis is a common trait of breast tumour cells

**DOI:** 10.1186/s13046-022-02514-y

**Published:** 2022-11-16

**Authors:** Ivan Mikaelian, Rudy Gadet, Mathieu Deygas, Philippe Bertolino, Anca Hennino, Germain Gillet, Ruth Rimokh, Sid-Ali Berremila, Michel Péoc’h, Philippe Gonzalo

**Affiliations:** 1 Centre de Recherche en Cancérologie de Lyon - Université Claude Bernard Lyon 1, UMR CNRS 5286, INSERM 1052, Centre Léon Bérard, 69373 Lyon, France; 2 Institut Curie, Paris Sciences et Lettres (PSL) Research University, Centre National de la Recherche Scientifique (CNRS), Unité Mixte de Recherche (UMR) 144, Paris, France; 3 Institut Pierre-Gilles de Gennes, PSL Research University, Paris, France; 4 Pathology department, UFR Medecine Saint-Etienne, CHU of Saint-Etienne, Saint-Etienne, France; 5 Biochemistry and Pharmacology department, UFR Medecine Saint-Etienne, CHU of Saint-Etienne, Saint-Etienne, France

**Keywords:** Human breast cancer, Breast epithelial cells, aerotaxis, EGFR, Migration, Invasion, Hypoxia, ROS

## Abstract

**Background:**

Aerotaxis, the chemotactism to oxygen, is well documented in prokaryotes. We previously reported for the first time that non-tumorigenic breast epithelial cells also display unequivocal directional migration towards oxygen. This process is independent of the hypoxia-inducible factor (HIF)/prolyl hydroxylase domain (PHD) pathway but controlled by the redox regulation of epidermal growth factor receptor (EGFR), with a reactive oxygen species (ROS) gradient overlapping the oxygen gradient at low oxygen concentration. Since hypoxia is an acknowledged hallmark of cancers, we addressed the putative contribution of aerotaxis to cancer metastasis by studying the directed migration of cancer cells from an hypoxic environment towards nearby oxygen sources, modelling the in vivo migration of cancer cells towards blood capillaries.

**Methods:**

We subjected to the aerotactic test described in our previous papers cells isolated from fresh breast tumours analysed by the Pathology Department of the Saint-Etienne University Hospital (France) over a year. The main selection criterion, aside from patient consent, was the size of the tumour, which had to be large enough to perform the aerotactic tests without compromising routine diagnostic tests. Finally, we compared the aerotactic properties of these primary cells with those of commonly available breast cancer cell lines.

**Results:**

We show that cells freshly isolated from sixteen human breast tumour biopsies, representative of various histological characteristics and grades, are endowed with strong aerotactic properties similar to normal mammary epithelial cell lines. Strikingly, aerotaxis of these primary cancerous cells is also strongly dependent on both EGFR activation and ROS. In addition, we demonstrate that aerotaxis can trigger directional invasion of tumour cells within the extracellular matrix contrary to normal mammary epithelial cells. This contrasts with results obtained with breast cancer cell lines, in which aerotactic properties were either retained or impaired, and in some cases, even lost during the establishment of these cell lines.

**Conclusions:**

Altogether, our results support that aerotaxis may play an important role in breast tumour metastasis. In view of these findings, we discuss the prospects for combating metastatic spread.

**Trial registration:**

IRBN1462021/CHUSTE.

**Graphical abstract: EGFR-dependent aerotaxis of primary breast cancer cells:**

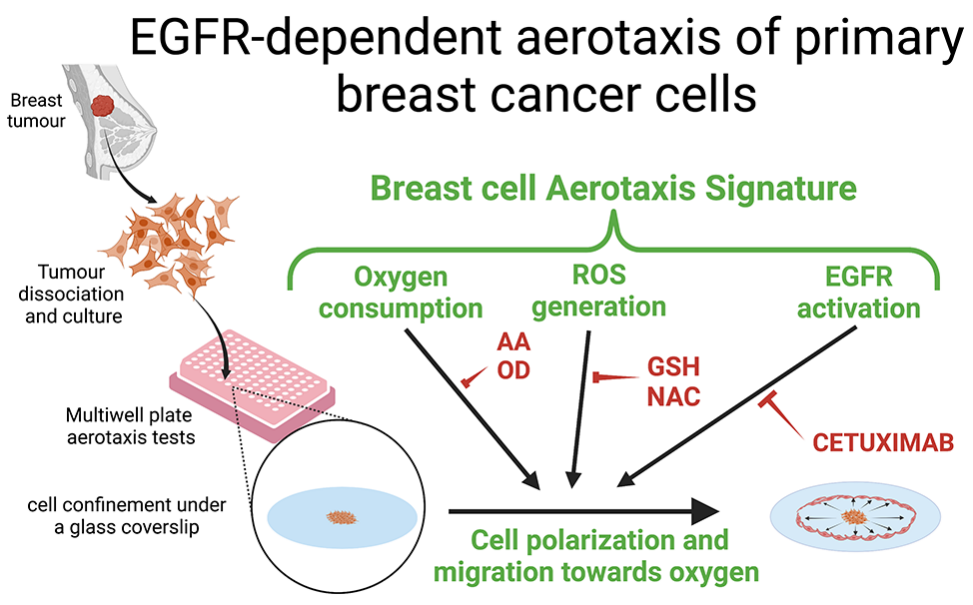

**Supplementary information:**

The online version contains supplementary material available at 10.1186/s13046-022-02514-y.

## Background

### Oxygen gradient origin

Oxygen gradients are widespread in the body and in the developing mammalian embryo. Among all of the nutrients carried by the bloodstream to cells, oxygen is the most limiting. Therefore, cells distant from a capillary must adapt to the lack of oxygen and rely on glycolysis for energy production instead of the highly efficient oxidative phosphorylation process. In tissues, depending on oxygen supply and on intensity of cell metabolism, median oxygen concentrations drop from 80 to 100 mmHg (103–129 µmol/L) in blood arteriolar capillaries to a median range of 24 mmHg (31 µmol/L) in the most efficiently perfused organs, such as the liver, kidneys, or the brain (organs also displaying the steepest oxygen gradient) and to ≤ 1% (9 µmol/L) in the most hypoxic ones [1]. These gradients fluctuate over short distances (tens of micrometres) representing the average distance between two capillaries. Even along these capillaries, and as shown by the so-called Krogh tissue cylinder model of 30 μm radius, normal values for the partial pressure of oxygen (pO_2_) reflecting a balance between arterial blood flow and tissue oxygen consumption, drop from 75 mmHg (97 µmol/L) at the inner part of the arterial end to below 10 mmHg (13 µmol/L) at the outer part of the venous end of the capillary [2, 3]. Hence, adjacent cells within the same tissue are exposed to very different oxygen concentrations and must therefore display very different metabolic properties.

### Aerotaxis in oxygen gradients

We recently demonstrated that mammalian epithelial cells from non-transformed breast epithelial cell lines, such as MCF10A, hMECt or MCF12A, have the potential to adapt to low oxygen concentrations by migrating towards higher oxygen gradients [4]. This particular taxis (ability to moves towards the source of stimulation) in MCF10A, MCF12A and HMECt cells depends on the activation of the Epithelial Growth Factor Receptor (EGFR or HER1), a major tyrosine-kinase receptor constitutively expressed in these cells and localised at the plasma membrane. Once activated by EGF binding, EGFR undergoes an auto-phosphorylation process leading to the activation of a wide variety of downstream signalling cascades. We demonstrated that the full activation of EGFR requires not only EGF binding but also ROS production by one or several unknown oxidases. As ROS production by oxidases depends on oxygen concentration, the activation of EGFR therefore relies on local oxygen availability. In the aerotaxis process, this unbalanced activation of EGFR between the leading and lagging edges of migrating cells is responsible for their polarization, and hence, for their directed migration.

### Aerotaxis as an alternative way to adapt to hypoxia

More remarkably, we showed that aerotaxis does not depend on the PHD / HIF pathway, the main process of adaptation to hypoxia [5]. In this pathway, the adaptation of cells, tissues and the whole organism to hypoxia depends on the regulation of HIF, a transcription factor that modulates the expression of hundreds of genes in humans. HIF is a protein with a fast rate of turnover in the presence of oxygen, its half-live being a few minutes. Indeed, oxygen is used as a substrate by the oxygen-sensing enzyme PHD to catalyse a proline hydroxylation within HIF. This post-translational modification triggers the recognition of HIF by the von Hippel-Lindau (VHL) ubiquitin ligase, leading to an accelerated degradation by the proteasome. When oxygen concentrations decrease, PHD-driven proline hydroxylation and subsequent polyubiquitylation and degradation of HIF are slowed down, leading to its accumulation and its translocation to the nucleus where it can exert its transcriptional regulatory activity. It is currently admitted that the K_M_ of PHDs for oxygen is around 70 mmHg (91 µmol/L), which is why HIF nuclear accumulation begins to be observed when the oxygen concentration drops below 30 mmHg (39 µmol/L), exceeding the oxygen concentration required to drive aerotaxis that we measured in another model [6]. Some examples of adaptations driven by HIF are given below. At the cellular level, the accumulation of HIF is responsible for the adaptation of cells to reduced oxygen availability by resorting to anaerobic glycolysis and coping with the resulting acidosis by increasing the expression of carbonic anhydrase IX. At the tissue level, HIF increases Vascular Endothelial Growth Factor (VEGF) transcription leading to neovascularization. At the level of the organism, sensing of hypoxia via HIF accumulation in the kidney results in erythropoietin (EPO) expression and thus to increased erythropoiesis and oxygen transport capacity. Aerotaxis offers another way to cope with hypoxia. Rather than adapting to or trying to combat hypoxia, aerotaxis steers cells towards higher oxygen concentrations. Since we recently reported its conservation in the *Dyctostelium discoidium* amoeba, aerotaxis probably constitutes a seemingly ancient and common feature of eukaryotic cells displaying respiration [6].

### Role of oxygen gradients

The influence or hypothetical role of these oxygen gradients remains to be fully addressed and characterized. Roles in morphogenesis have been proposed as well as an involvement of aerotaxis during mammalian embryogenesis, including in placentation, organ and limb development, or stem cell differentiation (see [7] for a review). In pathological situations such as cancer, and because of the considerable metabolic activities of tumours and their deficient vascularization [8], it is tempting to attribute a role to aerotaxis in the metastatic diffusion of cancer cells. Indeed, aerotaxis could set in motion and steer cancer cells to blood capillaries carrying oxygen, facilitating their dissemination into the bloodstream and, from there, throughout the body to foster tumour progression and metastasis.

### Object of this study

Having extensively described the directed migration of immortalized non-cancerous mammary cell lines, such as MCF10A, HMECt or MCF12A, towards oxygen gradients [4], our current objectives were to (i) investigate whether aerotaxis was also a property of breast cancer cells, and (ii) to assess whether the aerotactic signature unveiled in untransformed breast cells could be detected in breast cancer cells. To test our hypothesis that aerotaxis may play a role in the dissemination of breast tumour cells in oxygen gradients surrounding the tumour, we performed aerotactic tests on cells freshly isolated from mammary tumours to check whether these cancer cells had retained their aerotactic potential during the transformation process. In our original cell confinement assay, aerotaxis was assessed at three different levels which make up the aerotactic signature: (1) requirement of cell respiration to create a self-generated oxygen gradient; (2) sensitivity to ROS to mediate the signal; (3) activation of EGFR as the final target. We also compared theses primary breast cancer cells with commonly available breast cancer cell lines.

## Methods

### Clinical specimen

Breast tumour samples were obtained by the Pathology department of the Saint-Etienne University Hospital (France) over a period of a year and had to meet the following criteria: (1) patients with no neoadjuvant chemotherapy or previous surgery; (2) a tumour size greater than 2 cm to ensure routine diagnosis and normal patient management as well as our own experiments; (3) a signed informed consent by the patients. Twenty breast tumours representative of the main subtypes and grades of breast cancer were thus collected in accordance with the guidelines of the European Network of Research Ethics Committees (EUREC) following European, national, and local laws. In France, the accredited ethical committee of the CHU Saint-Etienne, France (Comité “Terre d’Ethique”; Institutional Review Board: IORG0007394) reviewed and approved this study (protocol IRBN1462021/CHUSTE; “AeroMove: l’aérotaxie, une cible thérapeutique contre la dissémination métastatique?“) in accordance with the “Loi Jardé du 5 mars 2012 relative aux recherches impliquant la personne humaine - RIPH” French law. This study was classified “RIPH 3 (little or no intervention; “without risk”). All participating patients signed an informed consent form approved by the responsible authority. The law provides that patients can withdraw their consent at any time. No biobank was created for the purposes of this study. No data other than gender, age and histological data were collected.

### Selection of tumour samples

For the twenty breast tumours collected, histological and immunochemical analyses were performed using standard procedures as previously described [9], leading to their classification according to the World Health Organization [10] and the Nottingham modification of the Scarff-Bloom-Richardson (SBR) histological score [11]. Among these tumours, the first three (T1, T2, T3) were used for the establishment of culture conditions and functional tests. In addition, one of the tumours from a male patient (T14) was discarded from the analyses as it did not grow in the medium that was selected from the preliminary trials. The clinical details for the remaining 16 female breast tumours are available in Table 1.

**Table 1:** Clinical details of breast tumours

Tumour Number	age (year)	TNM ^(1)^	Cancer type [10]	Grading SBR ^(2)^	Tumour size (cm)	Volume (cm3)	Mitotic activity ^(3)^	Ki67 ^(4)^	HER2 ^(5)^	ER ^(6)^	PR ^(7)^
**T4**	65.7	pT2 N2 Mx	Pleomorphic invasive lobular carcinoma + NST^(^*^)^	3 (D3, A3, M2)	5 × 3 × 3	45.0	4.0	30%	-	-	-
**T5**	66.9	pT2 N1 Mx	NST^(^*^)^	3 (D3, A3, M2)	2.7 × 2.5 × 2	13.5	4.6	/	+	+	+
**T6**	60.3	pT2 m N1	NST^(^*^)^	1 (D2, A2, M1)	3.7 × 3.5 × 2	26.22	1,0	15%	-	+	+
**T7**	78.6	pT2 N1 Mx	NST^(^*^)^	3 (D2, A3, M3)	2.2 × 1.7 × 1.8	6.7	8.3	30%	-	+	+
**T8**	35.9	pT2 N0 (sn) (mol)	NST^(^*^)^	3 (D3, A3, M3)	2.3 × 1.8 × 1.6	6.6	11,0	35%	-	+	+
**T9**	46.6	pT2 N1	NST^(^*^)^	1 (D1, A2, M1)	2.1 × 1.9 × 1.4	5.6	1.2	8%	-	+	+
**T10**	48.4	pT2 N1 mi (sn) (mol+)	NST^(^*^)^	2 (D3, A2, M2)	2.5 × 2 × 2.3	11.5	3,0	10%	-	+	+
**T11**	73.1	pT2 N1 Mx	NST^(^*^)^	2 (D3, A2, M2)	3.5 × 3 × 3.2	33.6	4.3	20%	-	+	+
**T12**	54.0	pT2 N2 Mx	Infiltrating duct carcinoma not otherwise specified (NOS) + Encapsulated papillary carcinoma	3 (D3, A3, M3)	2.7 × 2.7 × 2.5	18.3	11,0	35%	-	+	-
**T13**	57.6	pT2 N1 mi (mol+)	Infiltrating duct carcinoma not otherwise specified (NOS) + invasive lobular carcinoma	3 (D3, A2, M3)	4.1 × 3.3 × 1.6	21.6	8.3	30%	-	+	+
**T15**	69.5	pT3 N0 sn (mol-)	Invasive lobular carcinoma	2 (D3, A2, M1)	11 × 7.5 × 6.5	500.5	0.7	12%	-	+	-
**T16**	78.8	pT2 N0 sn (mol)	Ductal carcinoma in situ (DCIS)	2 (D2, A2, M2)	2.5 × 1 × 1.7	4.3	4.2	15%	-	+	+
**T17**	45.3	pT2N1mi(sn) (mol+)	NST^(^*^)^	2 (D3 ,A2 ,M1)	2.7 × 2.1 × 2.1	11.9	2,0	15%	-	+	-
**T18**	80.3	pT2N1mi (sn)(mol+)	Pleomorphic invasive lobular carcinoma	3 (D3, A3, M2)	2.2 × 2.1 × 1.4	6.5	6.3	25%	-	-	-
**T19**	45.9	ypT3N0	Invasive lobular carcinoma	2 (D3, A2, M1)	6.5 × 2.3 × 6.5	97.2	1.3	18%	-	+	+
**T20**	44.2	pT2NOsn(mol)	NST^(^*^)^	3 (D3, A2, M2)	2.1 × 2 × 1.8	4.6	4,0	15%	-	+	+

(1) Classification 2017 (American Joint Committee on Cancer (AJCC). AJCC cancer staging manual. 8th ed. (2017) New York, NY: Springer in WHO classification of Tumours − 5th edition- 2019.

(2) Scarff-Bloom-Richardson (SBR): D: differentiation; A: anisokaryosis; M: mitosis [11].

(3) Mitosis number / mm^2^

(4) Ki67 immunostainning with Dako Mib1.

(5) HER2 immunostainning with Dako A0485 and FISH confirmation (+ if overexpression = 2).

(6) Oestrogen Receptor expression with Dako Sp1.

(6) Progesterone Receptor expression with Dako PqR636.

^(*)^ NST: Invasive Breast Carcinoma of No Special Type (formerly: Invasive ductal carcinoma)

### Purification and amplification of tumour cells from breast cancer fragments

Fresh breast tumour samples (< 200 mm^3^) collected by the pathologists of the Pathology department of the Saint-Etienne University Hospital, Saint-Etienne, France, were resected at the centre of the tumour mass and stored in MACS Tissue Storage Solution (Myltenii Biotech, Germany) for 12 to 72 h at room temperature for transport to the Cancer Research Centre of Lyon (CRCL) laboratory. There, tumour fragments were mechanically and enzymatically dissociated with collagenase I (0.1 mg/mL, Merck USA) in phosphate buffered saline (PBS). Dissociated cells were collected by 2 min centrifugation at 1,200 rpm, filtrated from debris on 70 μm mesh filters, resuspended in 1 mL of Red Blood Cell Lysis Buffer (Roche, Germany) for 5 min at 4 °C to eliminate erythrocytes, and finally plated in 6-well plates in the appropriate medium. Growing breast cancer cells in 2D is challenging. At first, we tested different dedicated breast cell media, but T1, T2 and T3 cells were unable to grow in MCF10A, HMECt or MECGM (Mammary Epithelial Cell Growth Medium, Promocell, Germany) media. We finally succeeded in growing tumour cells in MHC medium, a medium inspired by Hans Clevers’s work on human breast cancer organoid culture and expansion [12]. MHC medium is based on Advanced DMEM/F12 (Thermofisher, USA) supplemented with 5% decomplemented foetal calf serum (Eurobio, France), 100 U/mL penicillin, 100 µg/mL streptomycin (Thermofisher, USA), GlutaMax (Thermofisher, USA), Hepes (Thermofisher, USA), 50 µg/mL Primocin (Thermofisher, USA), 0.5 µg/mL hydrocortisone (Merck, USA), 1X B27 supplement (Thermofisher, USA), 1.25 mM N-Acetylcysteine (NAC, Merck, USA), 5 mM Nicotinamide (Sigma-Aldrich, USA), 0.5 µM A83-01 (Bertin Pharma, France), 5 µM Y-27,632 (Bertin Pharma, France) and 5 ng/mL EGF (Peprotech, USA). After growing cells for 1–2 passages in MHC medium, EpCAM + epithelial cells were purified from fibroblasts and immune cells by magnetic immunopurification on MACS columns (Myltenii Biotech, Germany). Tumour cells could be amplified for at least 6 to 8 passages before a decrease in growth potential was observed. This limited time frame has nevertheless proved sufficient to characterize these epithelial cells and to address their aerotactic potential.

### Drugs and inhibitors

The following drugs and inhibitors were purchased from Merck (USA): N-acetyl-L-cysteine (NAC), reduced glutathione (GSH), and antimycin A (AA). Oligomycin D (OD) was ordered from Enzo life sciences (USA) and cetuximab (Erbitux) was obtained from Merck Biopharma (Germany).

### Description of the aerotactic assay

**Fig. 1:**
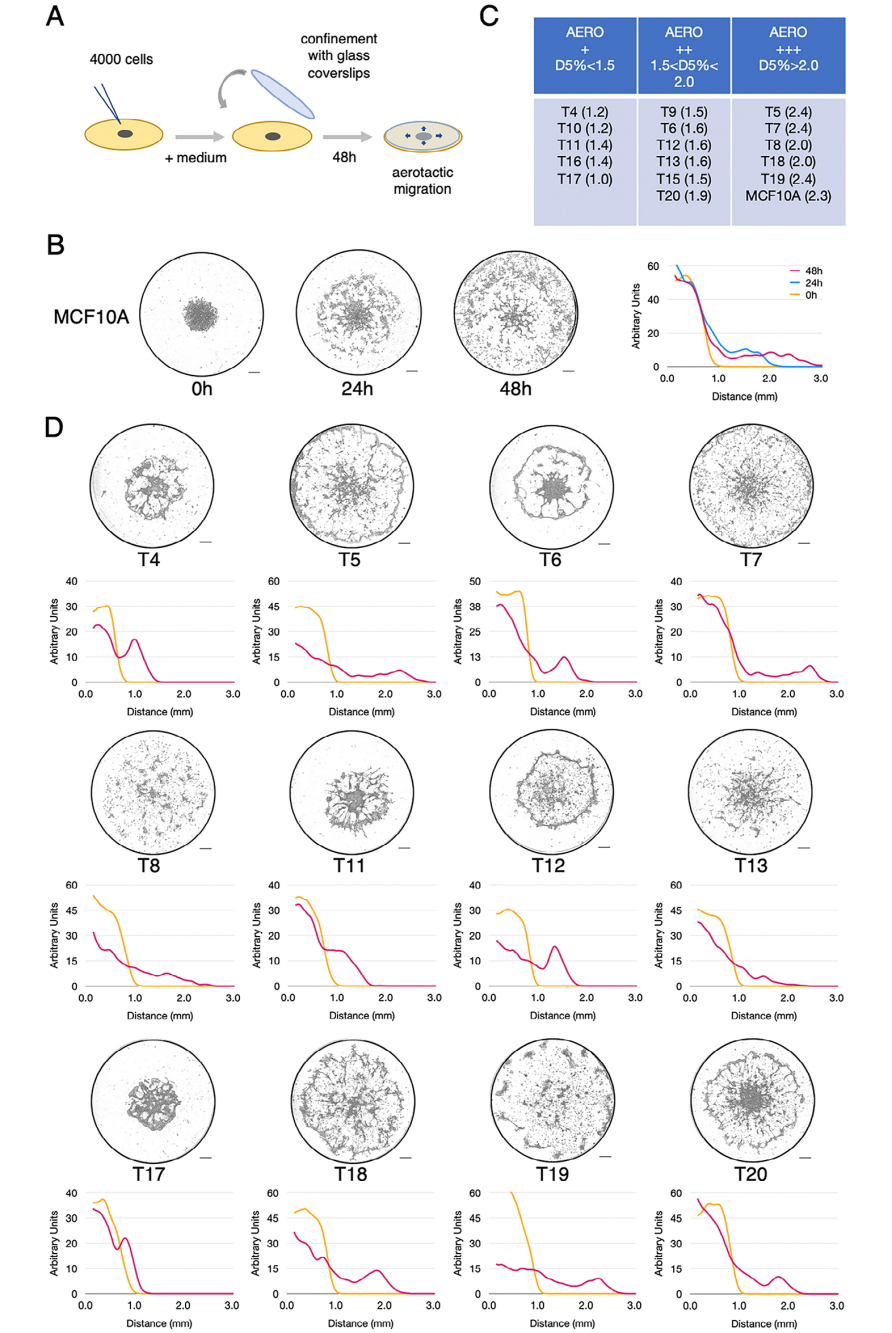
Migration of breast tumour cells in the aerotactic assay. **(A)** Schematic representation of the aerotactic migration assay. **(B)** Radial aerotactic migration from the central cluster of untransformed MCF10A cells in a 96-well plate over 24 and 48 h. The graph on the right corresponds to the distribution of MCF10A cells from the centre of the well (in mm) at 0 h (yellow), 24 h (blue) and 48 h (carmine) after confinement (mean of three experiments). The Y-axis represents cell-density in arbitrary unit as described in the Methods section. **(C)** Classification of tumour cells relative to MCF10A according to their speed of migration in the aerotactic test. D5% is the minimum distance travelled by the fastest 5% cells (see Methods). **(D)** Representative images taken at 48 h corresponding to the aerotactic migration of T4, T5, T6, T7, T8, T11, T12, T13, T17, T18, T19 and T20 tumour cells subjected to the aerotactic assay. Cell distribution from the centre of the well at 0 h (yellow) and 48 h (carmine) is indicated as graphs (mean of three experiments). (See also Figure S1 for T9, T10, T15 and T16 cells). Y-axis scale is expressed in arbitrary unit. Scale bars, 0.5 mm

The aerotactic assay used in this study was modified from our previously described methodology (Fig. 1A) [4]. To accelerate the read-out and reduce the number of cells required, it was scaled-down to a 96-well plate format, the confinement being performed under 6 mm in diameter glass coverslips (Trajan, Germany). More precisely, breast tumour cells or MCF10A cells used as control were resuspended in their appropriate culture medium at a concentration of 4,000 cells/µL and spotted as 1 µL drops at the centre of wells in 96-well plates. Plates were incubated at 37 °C for 5 h in a humidified atmosphere to allow cells to adhere before wells were gently filled with 200 µL of fresh medium. Medium was optionally supplemented with drugs or inhibitors at the indicated concentration to assess the existence of the aerotactic signature. This signature consists in the inhibition of the outwards directional migration of the cells at the periphery of the cell cluster by applying (1) inhibitors of the respiratory chain and oxidative phosphorylation using antimycin A (1 µM) or oligomycin D (0.5 µM); (2) ROS inhibitors such as GSH (10 mM) or NAC(10 mM); (3) inhibition of EGFR activation by cetuximab (12.5 and 25 µg/mL). Aerotaxis migration was triggered by confining cells under the glass coverslips dipped in culture medium at t_0_ and monitored by time-laps imaging using an Incucyte Zoom microscope (Sartorius, USA). Control experiments were performed in non-confined conditions.

### Analysis of cell migration

Extents of cell migration were analysed from time-lapse experiments performed with the Incucyte Zoom in 96-well for 48 h. To graphically analyse the extent of cell migration, whole well images obtained from the Incucyte were processed with imageJ as follows. Images were first cropped, then processed to reduce background and increase the signal to noise ratio. The coordinates of the spot’s centre corresponding to the barycentre of the image was computed with ImageJ. Migration analysis was then performed by compiling and smoothing the results of 30 radial “plot profiles” starting from the centre of the spot and plotting the mean signal intensity obtained against the distance from the centre of the spot. The X-axis scale of the resulting graphs are given in mm and labelled “Distance (mm)”. The Y-axis represents an estimate of cell density obtained through ImageJ processing of Incucyte images and is expressed in arbitrary units.

D5% is a calculated value indicative of the extent of cell migration. It represents the distance from the centre (in mm) which encompasses 95% of the signal obtained from the cells. When possible, the experiments were repeated three times to compare the conditions. While investigating the sensitivity of the different cell lines to EGF stimulation, a Student t-test was performed to assess whether or not D5% was significantly different. This statistical analysis proved useful when differences in cell migration were not obvious.

### Analysis of aerotactic and intrinsic cell motilities

Characteristics of cell motility were analysed from time-lapse experiments performed with the Incucyte Zoom in 96-well ImageLock plates for 24 h and 30 min intervals. For each condition, we manually tracked 50 cells from five independent experiments using the ImageJ software. Trajectories were represented as XY graphs. Cell migration parameters were calculated with an in-house developed Excel macro (Microsoft, USA). The total distance travelled consisted of the sum of displacements of one cell over 24 h. The relative distance travelled by a given cell is the distance separating its first and last position. Directionality is the ratio of the relative distance to the total distance travelled. Directionality tends towards zero if cells move randomly, and towards one if cells continuously move in the same direction. Cell speed is the ratio of total distance travelled to time. Intrinsic cell motility was analysed in the same way, except that the cells were not confined but seeded at 5% confluence, and were thus able to move randomly.

### Aerotaxis invasion of Cultrex basement membrane extract

Cultrex Basement Membrane Extract (BME) is an extracellular protein mixture secreted by Engelbreth-Holm-Swarm (EHS) mouse sarcoma cells (R&D Systems, Bio-Techne, USA). This hydrogel is a natural extracellular matrix (ECM) similar to Matrigel that mimics the complex extracellular environment. It is liquid at 4 °C and forms a gel at 37 °C. To address the potential of cells to perform aerotaxis invasion within ECM, we proceeded as described previously, but, following adhesion of cells, spots were immersed in 4 µL of 100% Cultrex at 4 °C before the glass coverslip was deposited to confine the cells within the ECM. 96-well plates were first incubated for 30 min at 37 °C to allow polymerisation of the hydrogel before 200 µL of fresh medium was gently poured onto the coverslip. Cell invasion within Cultrex was followed by time-lapse microscopy for 60 h with the Incucyte Zoom. Cell invasion was analysed as indicated above for cell migration.

### Culture of cell lines

The mammary epithelial cell line MCF10A, a spontaneous immortalised but non-transformed cell line, was obtained from the American Type Culture Collection. These cells were grown in DMEM/F12 medium (Thermofisher, USA), supplemented with 5% horse serum (Thermofisher USA), human recombinant EGF (10 ng/mL, Peprotech USA), insulin (Novorapid,10 µg/mL, Novo Nordisk Denmark), cholera toxin (100 ng/mL, Merck USA), hydrocortisone (0.5 µg/mL, Merck USA), penicillin (100 U/mL, Thermofisher USA) and streptomycin (100 µg/mL, Thermofisher USA).

Breast tumour cell lines; BT-20, CAL-51, CAMA-1, MDA-MB-157, MCF7, MDA-MB-231, Hs578T, SK-BR-3, BT-549, BT-474, MDA-MB-453, MDA-MB-361, MDA-MB-468 were grown in the following breast cancer (BC) medium; Advanced DMEM/F12 (Thermofisher, USA) supplemented with 10% foetal calf serum (Eurobio, France), 100 U/mL penicillin, 100 µg/mL streptomycin (Thermofisher USA), 0.5 µg/mL hydrocortisone (MERCK), GlutaMax (Thermofisher, USA), 25 mM Hepes (Thermofisher, USA), Non Essential Amino Acids (Thermofosher, USA), human recombinant insulin (10 µg/mL, Novorapid, Novo Nordisk Denmark). We first grew each of these cell lines in the culture medium recommended by the ATTC. However, in some cases and as described in the ATCC handling information, there was significant mortality in these media, with floating dead cells interfering with the experimental monitoring or the migration of adherent cells. We then switched to the BC medium which was superior or at least equivalent for all breast tumour cell lines, except T47-D. This led to an improvement in cell growth and a reduction in mortality. Moreover, the harmonization of the experimental conditions made it possible to carry out comparisons. T47-D were grown in RPMI supplemented with 10% foetal calf serum, 100 U/mL penicillin, 100 µg/mL streptomycin (Thermofisher, USA), 10 µg/mL of human recombinant insulin (Novorapid, Novo Nordisk Denmark). Human recombinant EGF (10 ng/mL, Peprotech USA) was added when indicated in the Figures.

### EGFR immunofluorescence

For immunofluorescence imaging, cells grown on glass coverslips were fixed in 4% paraformaldehyde, washed three times with PBS, permeabilised with 0.5% Triton X-100 in PBS for 10 min and washed again three times with PBS. Coverslips were then blocked with 3% BSA in PBS for 1 h, incubated with the mouse monoclonal anti-EGFR antibody (Santa Cruz USA, #sc-101; dilution: 1/100), washed three times with PBS, stained with rabbit anti-Mouse IgG (H + L) (Cross-Adsorbed Secondary Antibody, Alexa Fluor 488 (Thermofisher USA, A11059, USA; dilution; 1/2000)), washed again three times with PBS, counterstained with Hoechst (1/5000) and finally mounted with a fluorescent mounting medium (Agilent, USA) under a glass coverslip. Images were acquired with a Zeiss LSM 880 confocal microscope (Zeiss, Germany).

### Visisens evaluation of local hypoxia

Hypoxia under confinement was monitored using the Visisens detector unit DUO1 (PreSens, Germany) and the associated AnalytiCal1 software as previously described [4]. Cells were spotted in a 12-well plate and a glass coverslip pre-coated by PreSens with the proprietary oxygen sensor (equivalent to SF-RPSu4 sensor foil) was used to confine the cell clusters. Local oxygen concentration close to the cells was then measured after 5 min by placing the detector unit under the plate. Visisens calibration was performed by exposing the SF-RPSu4 sensor to ambient air (21% oxygen; 159 mmHg) or to a saturating Na_2_SO_3_ solution for 10 min (0% oxygen).

## Results

### Aerotactic migration is a common feature of breast cancer cells

Aerotactic potential of tumour cells from early passages was assessed using the confinement assay described previously and schematised in Fig. 1A [4]. Four thousand cells were plated as a 1 µL drop of a mean radius of 1.3 ± 0.1 mm in 96-well plates and confined after adhesion under 6 mm in diameter glass coverslips (see the Methods section). Due to mitochondrial respiration, hypoxia rapidly builds up under the coverslips, which generates an oxygen gradient at the periphery of the cell cluster. We previously showed that cells located within this self-generated oxygen gradient undergo a rapid and directional outward migration to reach regions of higher oxygen concentrations [4]. As cells migrate and continue to breathe, the gradient is translated outward, perpetuating the oxygen gradient at the cell migration front. This phenomenon is reproduced here in Fig. 1B and video 1, which show the aerotactic migration of non-transformed MCF10A cells subjected to the aerotactic test. To evaluate and compare aerotaxis migration of cells freshly isolated from breast tumour biopsies (passages P2-P6 depending on the cell growth rate), we plotted cell distribution along the radius of the spot at 48 h and calculated the D5% as the greatest distance from the centre reached by at least 5% of the cells in 48 h (see the Methods section) (Fig. 1C). Remarkably, the outward directional migration characteristic of aerotaxis was observed for all breast cancer tumours (Fig. 1D, Sup. Figure 1, and videos 2 & 3 showing T5 and T7 tumour cell migrations as examples). This consistency was striking despite the fact that travelled distances were variable. Tumours were ranked according to untransformed MCF10A breast cells (D5% >2 mm) with some cell populations migrating faster e.g. T5, T7, T19 while others, e.g. T4, T10, T11, T16 or T17 were much slower. The extent of migration of cells representative of these categories are shown in Fig. 1C.

### Aerotaxis efficiency is closely correlated with intrinsic cell velocity

**Fig. 2:**
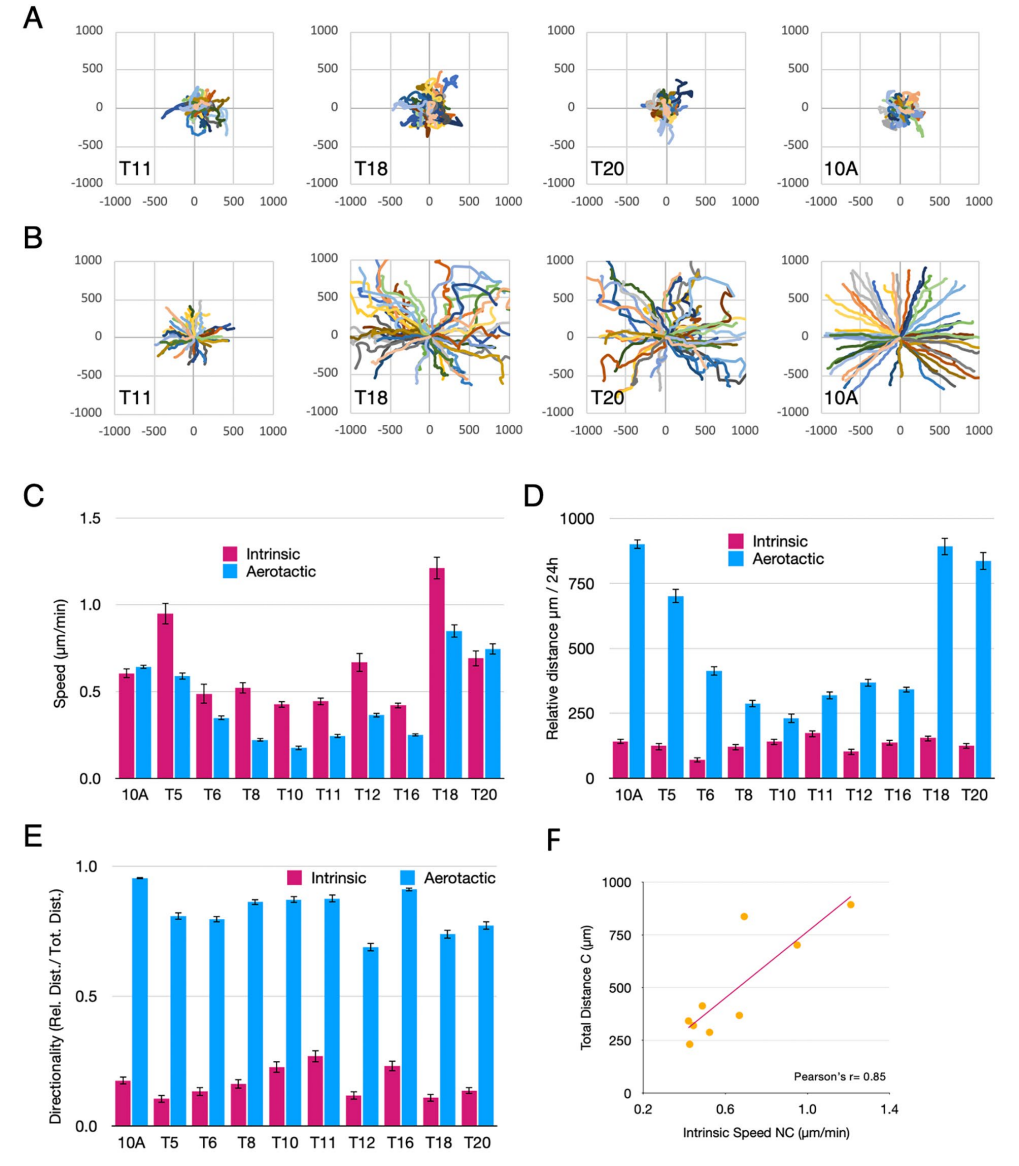
Comparison of aerotaxis versus intrinsic migration of breast cancer cells. 24 h trajectories of 50 representative cells per condition followed as XY plots (scales in µm) of the indicated cells either **(A)** plated at 5% confluency (intrinsic migration) or **(B)** subjected to the aerotactic assay (see also Figure S2 for T5, T6, T8 T10 and T16 cells). **(C)** Mean values of speed ± SD (in µm/min) of the indicated cells (10 A stands for MCF10A). **(D)** Mean relative distance of migration ± SD (in µm) of the indicated cells at 24 h. **(E)** Mean directionality ± SD (no unit) is given for aerotaxis (blue) and intrinsic (carmine) cell migration. **(F)** Graphical representation of the intrinsic speed of tumour cells as a function of the relative distance travelled in the aerotactic test. Pearson’s correlation coefficient is given. (All of the data displayed are representative of at least 3 independent experiments)

The aerotactic migration rate depends on the one hand on the directionality of the cells, i.e. their ability to detect and follow the oxygen gradient, and on the other hand, on their intrinsic velocity. To further analyse the observed differences in aerotaxis between tumours, and to better understand whether certain cell populations were more skilled than others at detecting and following oxygen gradients, we measured the intrinsic velocity of a selection of tumour cells by tracking isolated cells plated at a low density over a 24 h period at 30 min laps-time intervals (Fig. 2 A and Figure S2). Under these conditions, individual cells migrated randomly in order to establish cell-to-cell contacts, a common trait of epithelial cells, before they become immobile and divide once they have formed a small cluster. This experiment revealed that cells isolated from breast tumour samples were highly motile compared to most cell lines [13] with a mean intrinsic cell speed of 0.65 μm/min (from 0.42 to 1.21; Fig. 2 C). However, a small relative distance was travelled (129 μm/48 h ± 28, Fig. 2D), since displacements were random with a low mean directionality (0.17 ± 0.06, Fig. 2E). Similarly, we tracked the aerotactic cells located at the periphery of cell clusters subjected to the confinement assay (Fig. 2B and Figure S2). The mean aerotactic speed of cancer cells was lower (0.42 μm/min, from 0.18 to 0.85) compared to MCF10A cells (0.64 μm/min) but it was clearly directed outward with a mean directionality of 0.81 (from 0.67 to 0.91) close to that of MCF10A cells (0.95). Accordingly, a long relative distance was travelled (mean distance of 488 μm/48 h; from 231 to 893) (Fig. 2B, 2C, 2D, 2E, and Figure S2). We also observed that the relative distance travelled by aerotactic cells was closely correlated with cell intrinsic velocity with a Pearson r value of 0.85 (Fig. 2 F). This indicated that the variability in the aerotactic velocity of the different tumour cells was essentially dictated by their intrinsic speed and not their ability to better sense and follow the oxygen gradient.

From these experiments, we concluded that all of the cancer cells isolated from breast tumours studied herein had a strong disposition for aerotactic migration.

### Aerotactic migration of tumour cells depends on mitochondrial respiration for the auto-generation of the hypoxic gradient

**Fig. 3:**
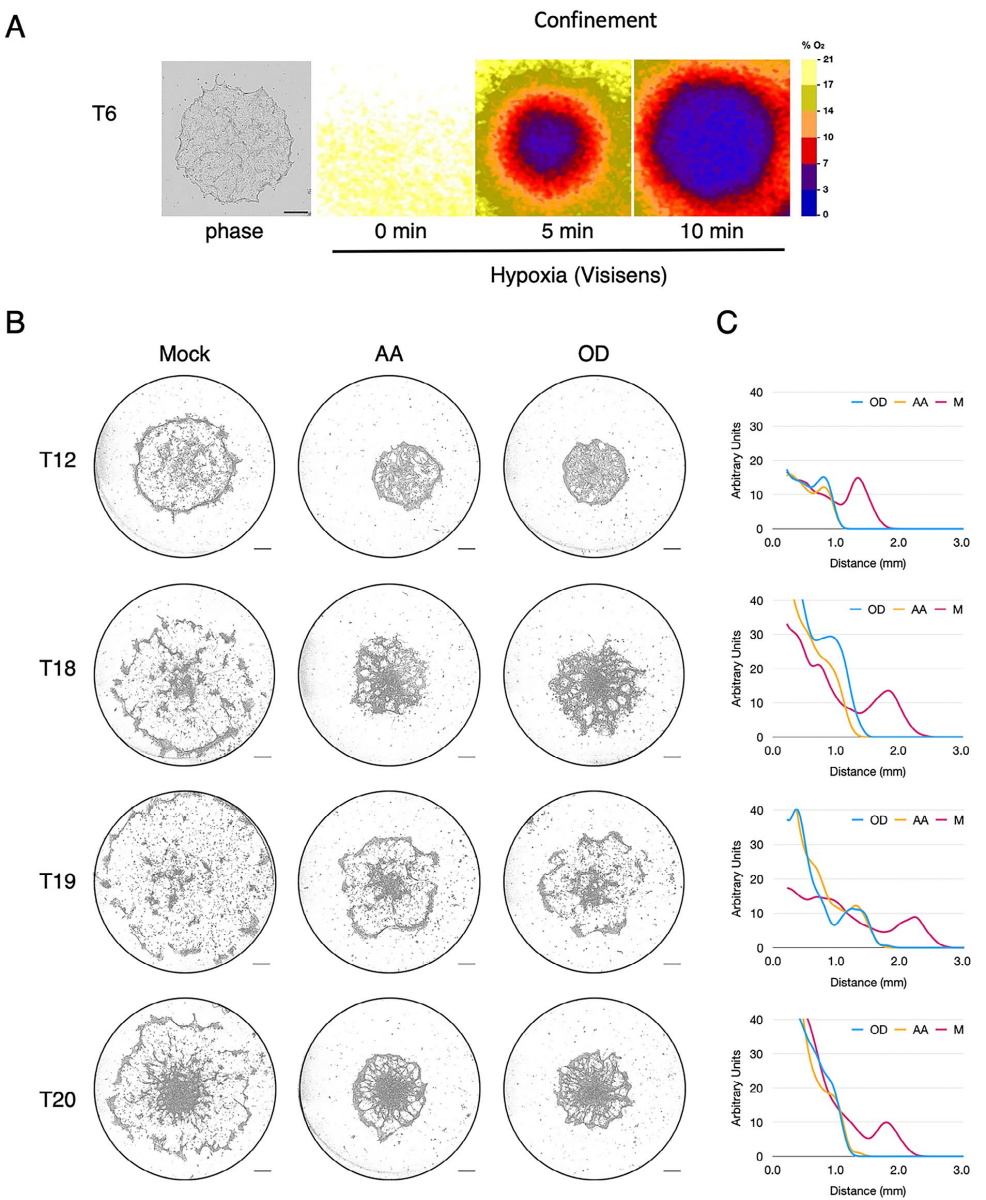
Breast cancer cell migration relies on a functional oxidative phosphorylation (OXPHOS) system. **(A)** Hypoxia generation by a cluster of T6 cells placed under confinement as visualised using the Visisens device (see Methods). Left panel: phase contrast image at 0 min. Right panel: detection of hypoxia generated at 0, 5 and 10 min after confinement. Scale Bar (0.5 mm). **(B)** Aerotactic migration at 48 h of the indicated tumour cells either untreated (Mock), treated with antimycin A (AA, 1 µM) or oligomycin D (OD, 0.5 µM). Scale bars 0.5 mm. See Sup. Figure 3 for the other tumour cells. **(C)** Cell distribution from the centre of the well at 48 h in the Mock condition (carmine, mean of three independent experiments), in cells treated with 1 µM AA (yellow) or 0.5 µM OD (blue). Y-axis scale is expressed in arbitrary unit

As previously described, mitochondrial respiration is required for aerotactic migration by creating an oxygen gradient at the periphery of the confined cell cluster [4]. To demonstrate that the outward migration of primary cancer cells also relies on the formation of such an oxygen gradient at the periphery of the cell cluster, we first measured the evolution of oxygen concentration within the T6 cell cluster with the Visisens apparatus (Fig. 3A). Hypoxia under these conditions developed very rapidly at the centre of the cell cluster together with the formation of an oxygen gradient at its periphery. We also tested on all primary cancer cells whether tumour cell migration under confinement could be inhibited by blocking cell respiration with inhibitors of the mitochondrial respiratory chain (Fig. 3B and Figure S3). Antimycin A (AA) and oligomycin D (OD) efficiently impaired aerotaxis of all tumour cells, suggesting that, similarly to MCF10A, the outward displacement observed with breast cancer cells was a *bona fide* aerotactic migration which requires functional mitochondrial respiration to generate a steep oxygen gradient at the edge of the cell cluster.

### Aerotactic migration of breast cancer cells is both redox sensitive and dependent on EGFR activation

**Fig. 4:**
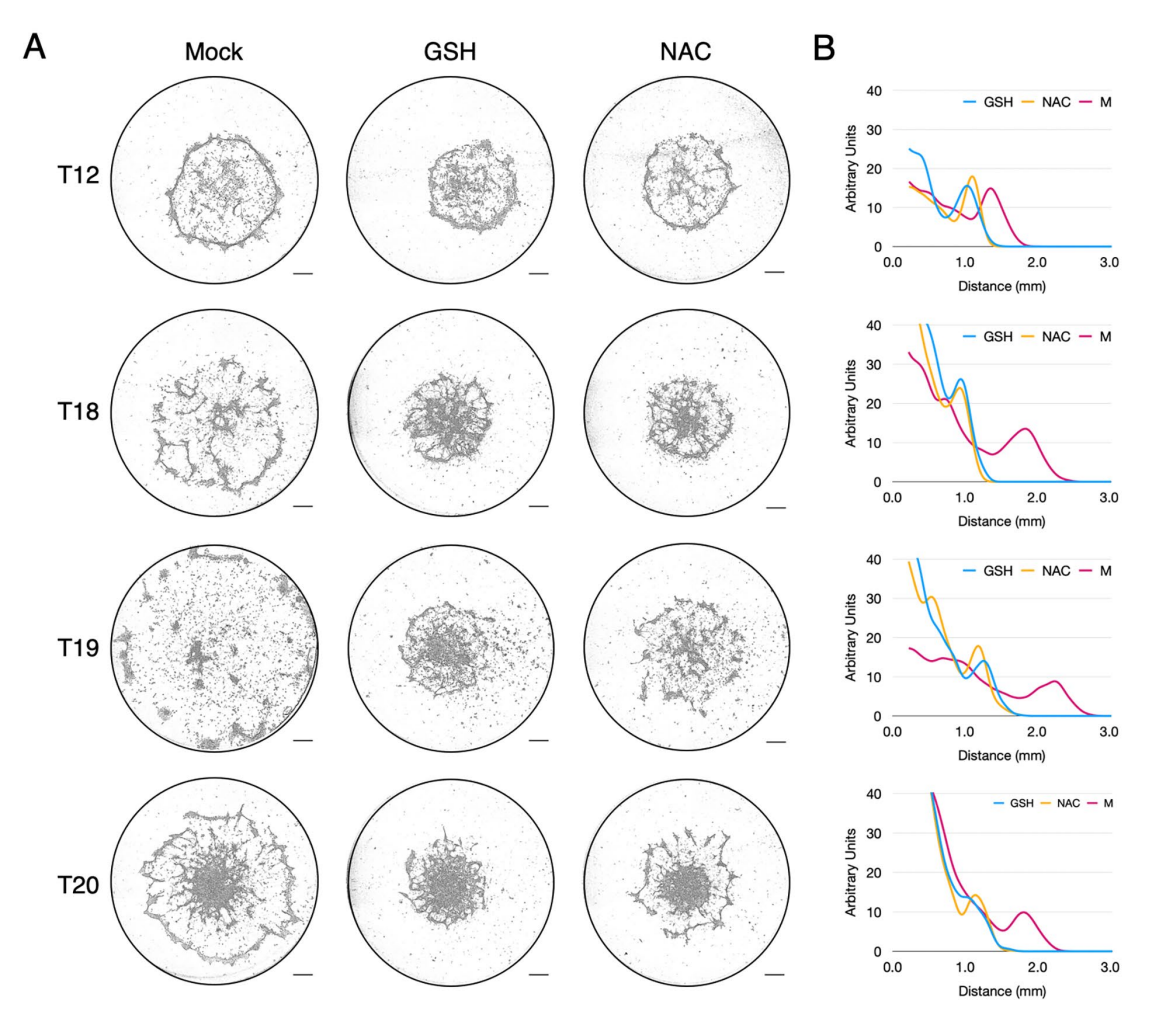
Aerotactic migration is impaired by antioxidants. **(A)** Aerotactic migration at 48 h of the indicated tumour cells either untreated (Mock), treated with 10 mM reduced glutathione (GSH) or 10 mM N-Acetyl Cysteine (NAC). Scale bars 0.5 mm. See Figure S4 for the other tumour cells. **(B)** Cell distribution from the centre of the cell cluster (in mm) at 48 h in the Mock condition (carmine, mean of three independent experiments), in cells treated with 10 mM GSH (blue) or 10 mM NAC (yellow). Y-axis scale is expressed in arbitrary unit

Another hallmark of aerotaxis in untransformed human breast epithelial cells is its dependence on ROS for its signalling. Indeed, we demonstrated that under confinement, an H_2_O_2_ gradient overlaps the oxygen gradient at the periphery of the cell cluster, owing to the fact that oxygen is the substrate for enzymes generating ROS [4]. To study the dependence of breast tumour cells on ROS to mediate aerotactic migration, we analysed the effect on migration under confinement of two ROS inhibitors, N-acetyl-L-cysteine (NAC) and reduced glutathione (GSH). Both compounds are substrates for enzymes that not only neutralize ROS, but also repair oxidative damage and signalling caused by ROS by alleviating their biological effects [14–16]. Both antioxidants efficiently impaired the migration of the breast tumour cells studied herein (Fig. 4 and Figure S4), indicating that breast cancer cell aerotaxis resembles that of untransformed breast cells as it is mediated by the biological effects of ROS, and thus indirectly by oxygen since ROS are generated from oxygen.

**Fig. 5:**
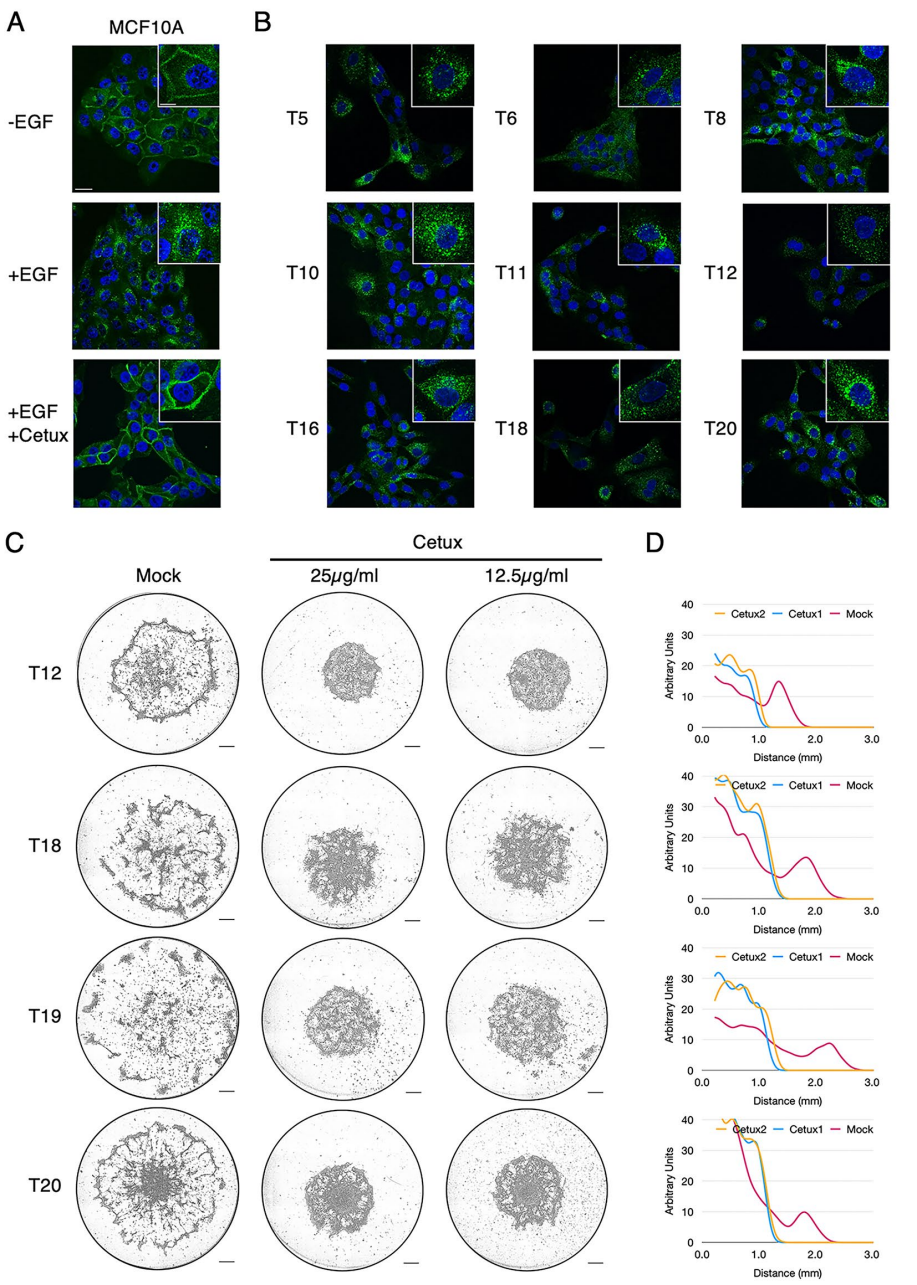
Aerotaxis of breast cancer cells is dependent on EGFR activation. **(A)** Immunofluorescence imaging of EGFR in MCF10A cells in the absence of EGF (-EGF), upon activation by EGF (+ EGF), or EGF + cetuximab, a monoclonal EGFR neutralising antibody (+ EGF + Cetux), **(B)** Immunofluorescence imaging of EGFR in the indicated tumour cells in their culture medium supplemented with EGF. Scale bars, 25 μm and 10 μm for high magnification views inserted in the upper-right corner of each image (x2.5). Steady-state EGFR (-EGF and + EGF/+Cetux conditions) is located at the plasma membrane, whereas it appears as intracellular dots upon activation (+ EGF condition). **(C)** Aerotactic migration at 48 h of the indicated tumour cells either untreated (Mock), treated with 25 µg/mL or with 12.5 µg/mL cetuximab (Cetux). Scale bars 0.5 mm. **(D)** Cell distribution from the centre of the cell cluster (in mm) at 48 h in the Mock condition (carmine, mean of three independent experiments), in cells treated with 25 µg/mL (blue) or with 12.5 µg/mL (yellow). Y-axis scale is expressed in arbitrary unit. See Figure S5 for the other tumour cells

In MCF10A mammary cells, EGFR was shown to be the biological target of ROS leading to aerotaxis [4]. Indeed, the complete phosphorylation and activation of EGFR by EGF, as for other tyrosine-kinases, requires ROS [17]. In the absence of EGF, EGFR is located at the plasma membrane. Following activation by its ligand, this receptor undergoes dimerization, phosphorylation, internalisation in clathrin-coated vesicles and finally degradation [18, 19]. To investigate the requirement of EGFR activation for aerotaxis, we first verified its expression by immunofluorescence (IF) in a selection of tumour cells (Fig. 5 A and 5B and Figure S5). Upon EGF withdrawal or by using cetuximab, a specific antibody inhibiting EGFR activation by EGF, EGFR seemed to be located at the plasma membrane of MCF10A cells but relocated following the addition of EGF to cytoplasmic vesicles to produce the dotted pattern typical of EGFR activation (Fig. 5 A). Immunofluorescence carried out on tumour cells showed that EGFR was well expressed and well activated in all tumour populations tested, as demonstrated by its specific vesicular pattern. To address EGFR dependency, we then challenged tumour cells with cetuximab, an EGFR neutralising antibody. Cetuximab treatment efficiently repressed aerotactic migration, demonstrating the strict requirement for a functional EGFR activation in this process (Fig. 5 C and 5D). This was also the case for T5, a tumour overexpressing HER2 (Figure S6).

Taken together, these results demonstrate that breast cancer cells have retained their ability to perform aerotaxis after transformation. In addition, for all of the tumours tested, aerotaxis seem to follow the cell signalling pathway initially described for untransformed breast cell lines, i.e. the oxygen-dependent redox regulation of EGFR.

### Aerotaxis triggers invasion of breast cancer cells in the ECM

Given that hypoxia and metastasis are typical features of breast tumours [20], the finding that aerotaxis is a common trait of primary breast cancer cells is of major interest regarding its possible involvement in metastasis. Indeed, steered by aerotaxis, malignant cells within primary hypoxic tumours are likely to migrate through the oxygen gradient to reach blood capillaries where oxygen concentrations are higher and to disseminate throughout the body to finally settle in distant organs.

**Fig. 6:**
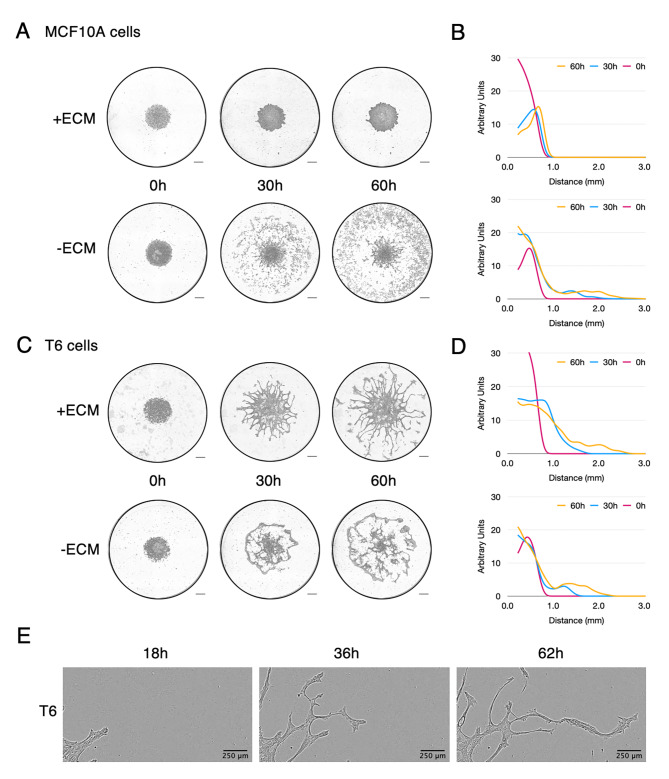
Aerotaxis triggers invasion of tumour cells through extracellular matrix (Cultrex BME). **(A)** Aerotactic assay of untransformed MCF10A cells within the ECM (+ ECM) compared to the standard condition (-ECM). **(B)** Cell distribution from the centre of the cell cluster (in mm) of MCF10A at 0 h (carmine), 30 h (blue) or 60 h (yellow) in ECM and standard conditions. Y-axis scale is expressed in arbitrary unit. **(C)** Aerotactic assay of T6 tumour cells within the ECM compared to the standard condition (-ECM). Scale bars in **A** and **C**: 0.5 mm. **(D)** Cell distribution from the centre of the cell cluster (in mm) of T6 tumour cells at 0 h (carmine), 30 h (blue) or 60 h (yellow) in the ECM and standard conditions (mean of three independent experiments). Y-axis scale is expressed in arbitrary unit. **(E)** Magnified image of the aerotactic invasion of T6 tumour cells within the ECM at 18, 36 and 62 h after confinement. Scale bars, 250 μm

However, the complex surrounding tumour microenvironment, including the extracellular matrix (ECM), is a strong barrier for cells to cross and we wondered whether aerotaxis would be a sufficiently powerful driving force for cells to invade the ECM. Therefore, we performed aerotactic experiments with cell clusters embedded within Cultrex Basement Membrane Extract (BME) and topped with a glass coverslip to generate the conditions of an aerotactic invasion. Cultrex BME is an extracellular protein mixture secreted by Engelbreth-Holm-Swarm (EHS) mouse sarcoma cells similar to Matrigel. As expected, untransformed MCF10A cells failed to invade this extracellular protein lattice since they lacked extracellular protein degradation enzymes (Fig. 6A and 6B, Video 4). In contrast, T6 tumour cells, efficiently migrated towards oxygen through the ECM (Fig. 6C and 6D, Video 5). However, aerotaxis within ECM differed from classical 2D aerotaxis. Cells migrated mainly as cohorts with leader cells perforating the matrix and opening tracks for the following cells in order to escape hypoxia (Fig. 6E and video 6). Therefore, our results suggest that aerotaxis is an efficient mechanism for tumour cells to invade the ECM, escape hypoxia, and reach areas better supplied in oxygen.

### Comparison with breast cancer cell lines

**Fig. 7:**
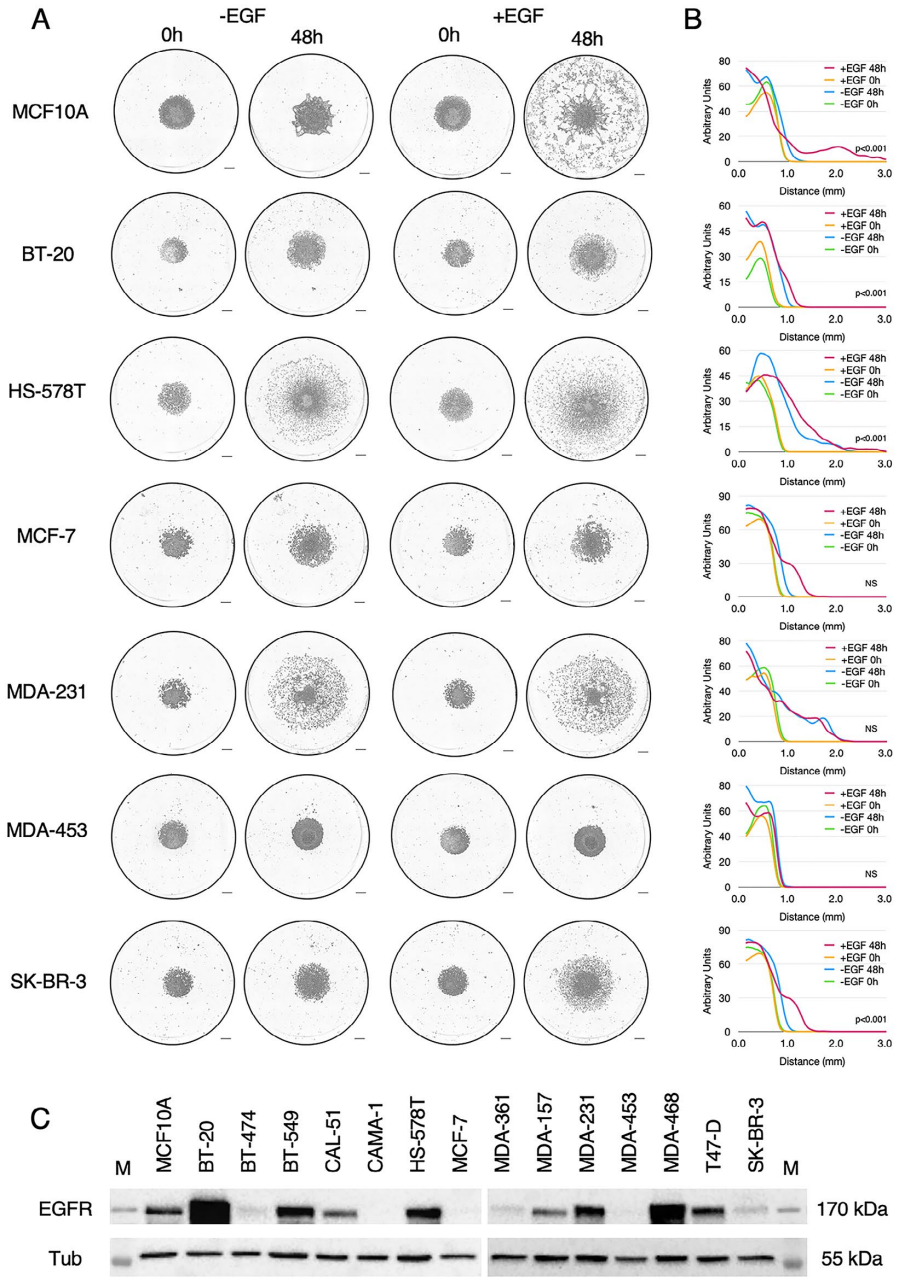
Migration of breast cancer cell lines in the aerotactic assay. (**A**) Representative images taken at 48 h of breast cancer cell lines subjected to the aerotactic assay in breast cancer medium (-EGF) or breast cancer medium supplemented with 10 µg/mL EGF (+ EGF). Scale bars, 0.5 mm. (**B**) Cell distribution from the centre of the cell cluster (in mm) at 0 h (green) and 48 h (carmine) in the -EGF condition and at 0 h (yellow) and 48 h (blue) in the + EGF condition is indicated as graphs (mean of three experiments). Y-axis scale is expressed in arbitrary unit. To assess the difference between the plus and minus EGF conditions at 48 h, a Student t-test was performed on D5% values. P-values are indicated within graphs. NS non-significant. (**C**) Western blot analysis of EGFR expression in breast cancer cell lines. Tubulin expression was used as a loading control. M stands for Molecular weight (PageRuler, Thermo). See also Figure S7 for other cell lines

The homogeneity of these results in breast primary cancer cells was unanticipated, given our previous experiments using cancer cell lines, and we did not expect that all cells from mammary tumours would exhibit aerotaxis, nor did we expect it to be EGFR-dependent in all cases. Therefore, we decided to test aerotaxis on a large number of readily available breast cancer cell lines. In an effort to harmonise the conditions of the aerotactic assay, we propagated these cell lines in a newly formulated medium as described in the Methods section, except for T47D cells which were grown in the ATCC recommended RPMI medium. Since all of these cell lines proliferated independently of EGF, they were first assayed in medium devoid of EGF. However, as we demonstrated above and previously [4] that aerotaxis of transformed or untransformed breast epithelial cells was EGFR dependent, their aerotactic potential was also simultaneously examined in medium supplemented with 10 ng/mL EGF (Fig. 7A, 7B and Figure S7).

**Fig. 8:**
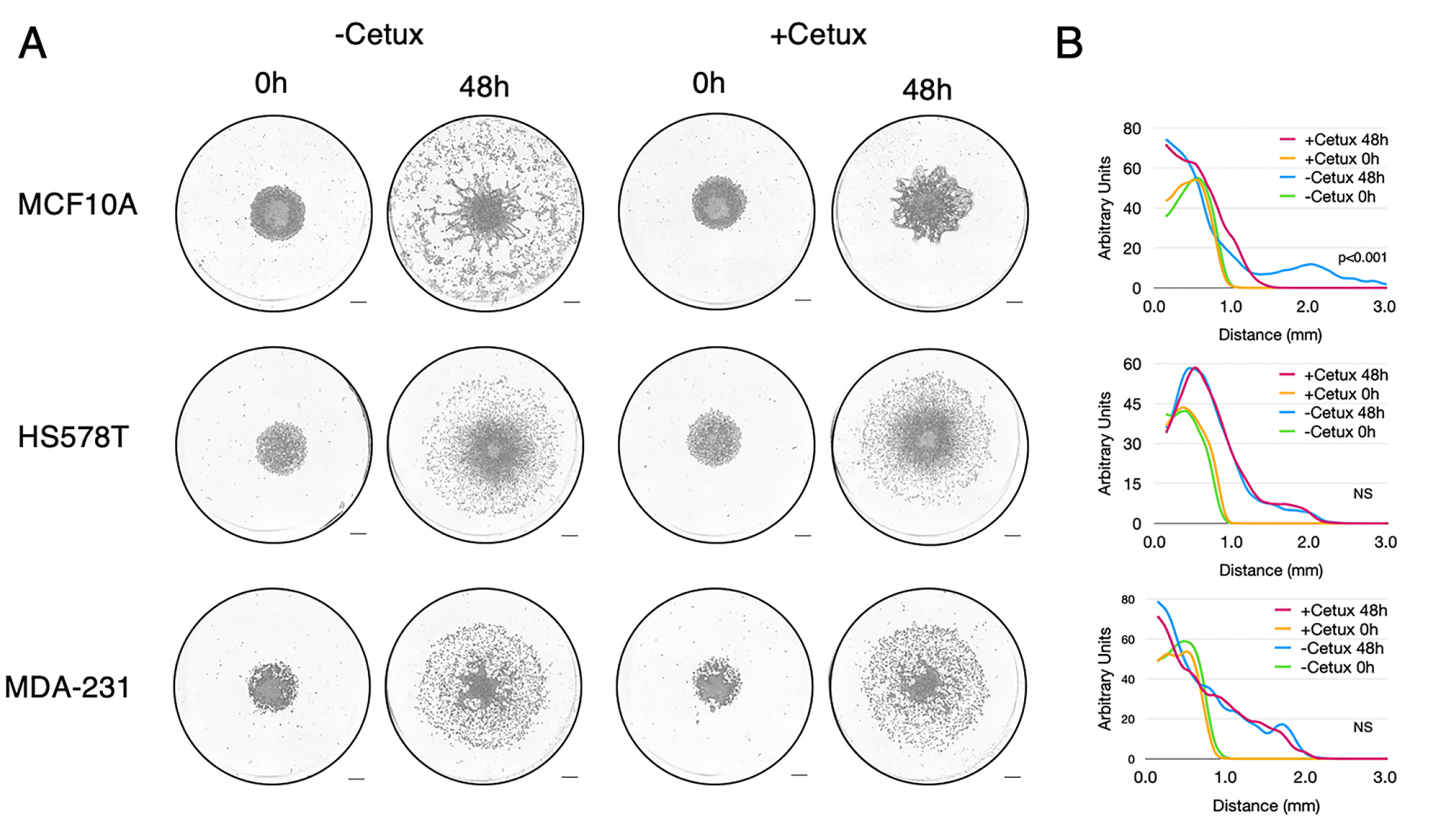
Aerotactic migration of Hs578T and MDA-MB-231 is independent of EGFR activation. **(A)** Representative images taken at 48 h of MCF10A, Hs578T and MDA-MB-231 cell lines subjected to the aerotactic assay incubated in MCF10A or BC medium supplemented or not with cetuximab as indicated. Contrary to MCF10A, aerotactic migration of both HS578T and MDA-MB-231 is not affected by cetuximab. Scale bars, 0.5 mm. **(B)** Cell distribution from the centre of the cell cluster (in mm) at 0 h (green) and 48 h (carmine) in the -cetuximab condition and at 0 h (yellow) and 48 h (blue) in the + cetuximab condition is indicated as graphs (mean of three experiments). Y-axis scale is expressed in arbitrary unit. To assess the significant difference between the plus and minus cetuximab conditions at 48 h, a Student t-test was performed on D5% values. The p-values are indicated within graphs. NS non-significant

Contrary to what was observed with the sixteen primary cancer cells isolated from fresh breast tumours, a strong heterogeneity was observed among breast cancer cell lines with four major different profiles (summarized in Table 2). Four cell lines namely CAMA1, MCF7, MDA-MB-453 and MDA-MB-361, were unable to perform aerotaxis whether EGF was present or not in the culture medium (Fig. 7A). Interestingly, this was correlated with the fact that these cells did not express EGFR as shown by Western blot analysis (CAMA1, MCF7, MDA-MB-453) (Fig. 7C) and as already described [21, 22], or were insensitive to EGF (MDA-MD-361 [23]) (Fig. 7C). The other ten breast cancer cell lines namely, SK-BR3, BT-549, BT-474, BT-20, CAL-51, MDA-MB-231, MDA-MB-157, T47D, Hs578T, and MDA-MB-468 all displayed aerotactic potentials. However, among them, five (SK-BR-3, BT-549, BT-474, BT-20, CAL-51) performed aerotaxis in a very similar way to the untransformed breast cell line MCF10A and the primary cancer cells extracted from the sixteen fresh breast tumours. Their aerotactic migration, although modest, was strictly dependent on EGF. It should be noted that it may seem surprising that BT-474 exhibits EGFR-dependent aerotaxis, albeit it expresses limited amounts of EGFR. However, it is known that in this cell line, the expression of the EGFR is induced by hypoxia [24]. Three other breast cancer cell lines, namely MDA-MB-231, MDA-MB-157 and T47D, underwent aerotaxis independently of EGF and EGFR. Indeed, although they expressed reasonable levels of EGFR, their aerotactic migration was not enhanced by EGF supplementation. The last pattern was displayed by Hs578T and MDA-MB-468, two EGFR-expressing cell lines which demonstrated EGF-independent aerotactic skills that were further increased by EGF. Blocking EGFR activation with cetuximab did not affect the EGF-independent aerotactic migration of these two cell lines, ruling out that they performed aerotaxis by secreting EGF, which would have rendered them independent on the addition of EGF to the culture medium (Fig. 8). Taken together, these findings suggest the co-existence of two independent molecular mechanisms able to trigger aerotaxis in breast cancer cell lines, one dependent on the classical EGFR activity, and one relying on another mechanism that remains to be identified. To decipher whether these four profiles could be linked to a molecular signature, we recapitulated the main characteristics of the tumour cells lines in Table 3. Seven of these fourteen mammary tumour cell lines are classified as Basal B (like MCF10A cells), two are Basal A, seven are Luminal. Among these cell lines, breast tumour cells migrating as MCF10A cells arose from all the subtypes. Specifically, three of these five tumour cell lines that performed EGFR-dependent aerotaxis were Basal B and two of the five were Luminal. All cell lines (4/4) that showed no aerotactic potential were Luminal. Two of the three cell lines that exhibited an EGFR-independent aerotaxis were Basal B and one of the three was Luminal. Finally, in the two cell lines that displayed aerotaxis independently of EGFR activation but stimulated by EGF, one was Basal A and the other was Basal B. Based on this analysis, the molecular subtypes of breast cancer cell lines do not seem to be linked to their aerotactic profiles.

**Table 2:** Summary of the aerotactic potential of breast cancer cell lines

		Aerotaxis					
**Profile**	**Cell lines**	**Medium - EGF**	**Medium + EGF**		**Medium (± EGF) + cetuximab**		**total EGFR**
**1**	**MCF10A**	**-**	**+++**	p < 0.001	(+ EGF): **-**	(p < 0.001)	**++**
	**SK-BR3**	**-**	**++**	p < 0.001	NI		**+**
	**BT-549**	**-**	**+**	p < 0.001	NI		**+++**
	**BT-474**	**-**	**+**	p < 0.001	NI		**±**
	**BT-20**	**-**	**±**	p < 0.001	NI		**+++**
	**CAL-51**	**-**	**±**	p < 0.05	NI		**++**
**2**	**CAMA1**	**-**	**-**	NS	NI		**-**
	**MCF7**	**-**	**-**	NS	NI		**-**
	**MDA-MB-453**	**-**	**-**	NS	NI		**-**
	**MDA-MB-361**	**-**	**-**	NS	NI		**±**
**3**	**MDA-MB-231**	**+++**	**+++**	NS	(- EGF): **+++**	NS	**++**
	**MDA-MB-157**	**+**	**+**	NS	ND		**++**
	**T47D**	**+**	**+**	NS	ND		**++**
**4**	**Hs578T**	**+++**	**++++**	p < 0.001	(- EGF): **+++**	NS	**+++**
	**MDA-MB-468**	**+**	**++**	p < 0.001	ND		**++++**

-, ±, +: Existence or not, and intensity of the aerotaxis

Column “Aerotaxis + EGF”: intensity of aerotaxis with EGF and statistical difference with condition -EGF.

Column “Aerotaxis + cetuximab”: intensity of aerotaxis in the presence of cetuximab in the medium ± EGF and statistical difference with the corresponding medium ± EGF.

p: result of the Student t-test on D5% values.

NS: non-significant.

ND: not determined (usually when migration was not fast enough to expect a significant difference).

NI: no interest when there is no migration to inhibit.

Column total EGFR: Intensity of EGFR expression observed by Western blot. For full results, see Figs. 7 and 8.

**Table 3:** Characteristics of the cell lines used in this article

Aerotaxis profile	Cell lines	Year of isolation	Source	Tumour Type [40]*	First description	Cell morphology	Karyotype (ATCC, [41], [42]	ER [40]*	PR [40]*	HER2 [40]*	Subtype [40]*	Subtype [43]	Tumorigenic in nude mice **
**1**	**MCF10A**	1984	PB	F	[44]	epithelial	48.XX.3-, 6p+, + 8, 9p+,+16 with focal deletions in 3p26.3 and 9p21.3 and focal amplification in 8q24.21 responsible for MYC amplification.	-	-	-	BaB	/	**-**
	**SK-BR-3**	1970	PE	AC	[45]	epithelial	Hypertriploid to hypotetraploid (modal number 80 ~ 84). Very numerous structural abnormalities. Three normal X chromosomes in most cells.	-	-	+	Lu	H	**+**
	**BT-549**	1978	PB	IDC/Pap	[46]	epithelial	Hypertriploid (modal number 78; range 73 ~ 80) and aneuploid (X chromosomes all abnormal).	-	-	-	BaB	TNB	**+** [47]
	**BT-474**	1978	PB	IDC	[46]	epithelial, patchy	Hypertetraploid (range 65 ~ 106) and aneuploid (XO usually). Numerous numerical and structural abnormalities.	+	+	+	Lu	LB	**+**
	**BT-20**	1958	PB	IDC	[48]	epithelial	Diploid cell line (modal number 49 ; range 43 ~ 53). Normal chromosomes N3, N4, N9, N13, N14, and X may be absent.	-	-	-	BaA	TNA	**+**
	**CAL-51**	1990	PE	AC	[49]	epithelial-like	Normal karyotype. PIK3CA mutation p.E542	-	-	-	TN	TNB	**+** [49]
**2**	**CAMA1**	1978	PE	AC	[50]	epithelial	Hypertriploid (modal number 80; range 68 ~ 83) with numerous rearrangements. Paired normal X chromosomes present in every cell.	+	- [40] ± [43]	-	Lu	LA	**+**
	**MCF7**	1975	PE	IDC	[51]	epithelial, loosely attached	Hypertriploid to hypotetraploid (modal number 82; range 66 ~ 87). 58 different rearrangements (31 numerical and 27 structural).	+	+	-	Lu	LA	**+**
	**MDA-MB-453**	1976	PCE	AC	[52]	epithelial	Hypo- to near-tetraploid (modal number 90; range 87 ~ 91 and aneuploid. Numerous numercial and structural abnormalities. Near-diploid population initially reported replaced by a tetraploid population.	-	-	- [40] + [43]	Lu	H	**+** [53]
	**MDA-MB-361**	1973	BM	AC	[52]	epithelial, loosely attached	Hyperdiploid (modal number 56; range 54 ~ 61) and aneuploid. Numerous structural and numerical abnormalities.	+	± [40] - [43]	+	Lu	LB	**+**
**3**	**MDA-MB-231**	1973	PE	AC	[54]	epithelial	Near triploid (modal number 64, range 52 ~ 68) and aneuploid. Numerous structural abnormalities.	-	-	-	BaB	TNB	**+**
	**MDA-MB-157**	1972	PE	AC	[55]	epithelial	Near triploid c(modal number 52 to 54; range 52 ~ 69) with 3 normal X chromosomes. Numerous structural abnormalities on most autosomes.	-	-	-	BaB	TNB	**+**
	**T47D**	1974	PE	IDC	[56]	epithelial	Hypotriploid (modal number 65; range 57 ~ 66). Very numerous numerical and structural abnormalities.	+	+	-	Lu	LA	**+** [57]
**4**	**Hs578T**	1977	PB	IDC	[58]	epithelial, stellar	Hypotriploid (modal number 59) with numerous rearrangements and abnormalities.	-	-	-	BaB	TNB	**+** [59] ***
	**MDA-MB-468**	1977	PE	AC	[13]	epithelial	Hypotriploid (modal number 64; range 60 ~ 67) and aneuploid (X, abnormal X). Numerous numerical and structural abnormalities.	-	-	-	BaA	TNA	**+**

**Source**: PB, Primary, Breast; PE, Pleural effusion; PCE, Pericardial effusion; BM, Brain metastasis. **Tumour type**: F, fibrocystic disease; AC, adenocarcinoma; IDC, invasive ductal carcinoma; Pap, papillary. **Subtype [**40**]: Lu, Luminal; BaA, BasalA; BaB, Basal B; TN, Triple negative**. *Except CAL-51 described in https://www.dsmz.de/collection/catalogue/details/culture/ACC-302. **Subtype** [43]: LA, luminal A; LB, luminal B; H, HER2 positive; TNA, Triple negative A; TNB, Triple negative B. **according to ATCC or ECAAC, except where a reference is cited. ***https://www.culturecollections.org.uk/products/celllines/generalcell/detail.jsp?refId=86082104%26collection=ecacc_gc

## Discussion

Hypoxia is a major feature of most tumours and it is known as a negative prognostic and predictive factor owing to its contribution to chemoresistance, radioresistance, angiogenesis, invasiveness, metastasis and resistance to cell death [25]. That hypoxia plays such an important role in such a diversity of processes is not surprising since HIF, the key transcription factor that accumulates during hypoxia, up- or down-regulates the expression of hundreds of genes [26]. We previously reported that untransformed human epithelial cells grown in a hypoxic environment were endowed with the ability to migrate towards oxygen [4]. This process, known as aerotaxis, or the ability of cells to sense and migrate towards oxygen, was first established 140 years ago by Engelmann for aerobic bacteria, although by a very different mechanism [27]. We unveiled that aerotaxis in breast epithelial cells is independent of the PHD-HIF pathway and that it relies on a post-translational mechanism mediated by the production of reactive oxygen species. In addition, we also demonstrated that these ROS were not by-products of the mitochondrial respiratory chain and that they were produced by non-mitochondrial oxidases. Aerotaxis is likely an ancestral process shared by most eukaryotes since we also described its existence in the amoeba *Dyctostelium discoidum*, which is devoid of a HIF ortholog [6].

From the discovery of aerotaxis in humans and the observation that hypoxia is a major driver of cancer progression and metastasis, it is tempting to infer that this mechanism could promote the metastatic process by guiding hypoxic cancer cells towards the blood capillaries that supply the tumours. Here, we report for the first time aerotaxis in primary cancer cells using a set of sixteen fresh breast tumours representative of all major breast cancer subtypes. The use of primary cells demonstrates that aerotaxis is not a property selected by cell culture but an intrinsic property of human epithelial cells which is retained throughout the tumour transformation process. However, the use of primary cells has limitations, in particular a risk of population drift following passages, with the selection of clones most capable of growing in vitro. Therefore, the amplification and the number of passages were restricted, and experiments not initially planned or replicates were more limited than with the cell lines. Our approach took these limitations into account and consisted in performing aerotactic tests, and in trying to inhibit it using a set of inhibitory compounds, in order to examine whether the so-called aerotaxis signature could be obtained. Aerotactic tests only require a few thousand cells which can be obtained from small fragments of primary tumours, with most of the tumour remaining available for routine diagnostic testing and patient management by pathologists.

This approach proved satisfactory in meeting our main objective which was to examine whether aerotaxis could be observed in tumour cells, and if so, whether it was widely shared or restricted to certain histological types. Our results demonstrate that all of the tumour cells studied are endowed with this property. Indeed, the cells of all primary breast tumours displayed an identical signature. 1) These cells migrated in a directional way towards oxygen. 2) This migration was directed towards oxygen since the use of inhibitors of the respiratory chain, which prevented the use of oxygen and therefore the in situ creation of hypoxia, inhibited aerotaxis. 3) This directional migration depended on ROS, since NAC and GSH that both impede the biological effects of ROS also inhibited aerotaxis. 4) Remarkably, all of the tumours studied including those that also overexpressed other types of receptor tyrosine kinases, such as HER2 (e.g. T5), depended on the controlled activation of EGFR for aerotaxis, indicating that EGFR plays a very specific role in cell polarization associated with directional migration. This last result was particularly striking for at least two reasons. First, aerotaxis can be observed in cells from tissues other than the breast such as HEK-293T (human embryonic kidney), which do not express EGFR and therefore do not depend on this receptor for aerotaxis [4, 28]. Second, EGFR is not described as a universal oncogenic driver in breast tumours, suggesting that, at least in some tumours, aerotaxis may be dependent on other signals. However, our results suggest that, in aerotaxis, EGFR is involved in a very specific pathway not shared by usual oncogenic drivers, including its closely related co-receptor HER2 with which it shares a strong homology.

The fact that aerotaxis itself is common to all primary tumours was striking because on the one hand, not all cancer cells were described as motile [13] and on the other hand, it was observed that transformed cells, while invasive, were not necessarily more mobile than their parental cell lines, the movements of some tumorigenic cells being more random [29, 30]. The originality and novelty of this finding was confirmed by the experiments with breast cancer cell lines, most of which had been cultured in laboratories for decades. Again, cell motility was highly variable depending on the cells, but unlike fresh breast tumour cells, four of the fourteen breast cancer cell lines were incapable of aerotactic migration. Interestingly, among the fourteen breast cancer cell lines, these four cell lines alone did not express EGFR or were insensitive to EGF activation [21–23], suggesting a specific role for this receptor. That aerotaxis may have disappeared in these cell lines together with EGFR expression may be the result of a gradual independence of EGF for cell growth in in vitro cell culture conditions over decades, a selection favoured by high mortality rates. However, the results obtained with the other ten breast cancer cell lines suggest a more complicated explanation. While five of the cell lines behaved the same way as MCF10A cells and the primary cancer cells from the sixteen primary breast tumours studied, their aerotaxis depending on the EGF activation of EGFR, three cell lines strongly expressing EGFR seemed to performed aerotaxis independently of EGF binding, and two cell lines seemed to only depend accessorily on the EGF activation of EGFR. Hence, in five of the fourteen cell lines, aerotaxis was EGFR-independent, albeit EGFR was significantly expressed. This demonstrates that aerotaxis can also be mediated by another pathway, at least for its end target, EGFR. This situation was reminiscent of that observed in HEK-293T cells, the prototypical epithelial cells that do not express EGFR but display aerotaxis and for *Dictyostellium* cells, in which no ortholog of EGFR has been found [4, 6]. An additional conclusion that can be drawn from this comparison between freshly isolated breast cancer cells and breast cancer cell lines, is that the latter, although readily available and suitable for in vitro culture, do not necessarily reproduce the original characteristics of the cells of origin. However, such a remark could also be extended to MCF10A cells, a common model of normal epithelial cells, but with its own limitations [31].

In breast cancer, the main therapeutic challenge is the treatment of metastases since localised tumours can be operated on. Our results clearly demonstrate that aerotaxis can lead breast cancer cells to invade a blood vessel, since its signalling only depends on the existence of an oxygen gradient, which occurs between tumour cells and the blood vessels that irrigate it. This observation is strongly reminiscent of that of Lewis et al. in which sarcoma cells responded to the hypoxic gradient by aggressively invading the matrix, and subsequently showed fast and long distance migration [32]. Inhibition of aerotaxis would therefore be a putative means of reducing metastatic spread. Of all the aerotaxis inhibitors tested, cetuximab, the EGFR inhibitor, was the most effective and probably the easiest to use. Indeed, it was effective in all tumour subtypes, including those overexpressing HER2, a tyrosine kinase receptor from the same family as that with which EGFR can heterodimerize [33]. Cetuximab also inhibited aerotaxis in the T18 triple negative breast cancer (TNBC), a type of breast cancer in which the therapeutic stake is the greatest once it has reached the metastatic stage. TNBC is a heterogeneous collection of breast cancers lacking expression of oestrogen receptor (ER), progesterone receptor (PR), and HER2 amplification, the targets which form the basis for major first line therapies in cancer. As a consequence, chemotherapy is the current standard-of-care for TNBC in the adjuvant, neoadjuvant, and metastatic settings. Patients with TNBC have a higher risk of local and distant recurrence, and metastasis is more likely to occur in the brain and lungs than in bone compared to other subtypes [34]. TNBC is initially highly sensitive to these chemotherapies but in many occurrences, resistance develops and metastases are highly lethal [35]. EGFR is frequently overexpressed in basal-like breast cancers, which comprises most TNBCs, and this overexpression is associated with metastasis progression and poor clinical outcome [36]. However, contrary to other types of cancers that express EGFR, such as pancreatic cancer, colorectal cancer or head and neck cancer where inhibition of EGFR was successful, attempts to evaluate the benefits of EGFR-inhibitors (both TKI and neutralising antibodies) in metastatic breast cancer have been disappointing [37]. The reasons for such an unexpected finding was likely that, while EGFR may be highly expressed in the original tumour, it is strongly downregulated (more than 1,000 times) in metastases by a yet unknown mechanism. This observation was reported for the first time in DU4475 (skin metastasis) and AIAb 496 (pulmonary metastasis). In these cell lines, cell growth was independent of EGF concentration, whereas in the T47D parental cell line, it was stimulated at low EGF concentrations (0.1 to 1 ng/mL) whereas it was inhibited over 10 ng/mL, a property that was shared by most cell lines that expressed EGFR [38]. Similar observations have been made in isogenic cell models and breast cancer tissues derived from female patients, with a strong correlation between EGFR expression and resistance to EGFR inhibitors [37]. Therefore, it is likely that EGFR inhibitors might only be of interest in preliminary stages to prevent the spreading of metastases from the primary tumour. A study in which this was the case (anti-EGFR used in neoadjuvant therapy before surgery in combination with a chemotherapy) highlights the benefit of including an anti-EGFR antibody (panitumumab) to increase the rate of complete pathological response (Chevallier classification, classes 1 + 2) for operable TNBC [39]. In this latter study, a better sensitivity was observed for tumours expressing high levels of EGFR and the safety profile was considered as overall manageable using prophylactic measures. Our study in which aerotaxis was observed in cells from all freshly collected breast tumours representative of the major types of breast tumours supports the use of cetuximab at an early stage of breast cancer progression.

## Conclusion

We can conclude that aerotaxis observed in untransformed breast cell lines is conserved in primary breast tumour cells, regardless of their subtype and therefore regardless of their pathway of neoplastic transformation. The mechanism of aerotaxis depends on the generation of ROS in response to hypoxia and EGFR for directed cell motility. We also demonstrate that oxygen is a sufficiently powerful attractant to guide tumour cells during the invasive process. We also show that aerotaxis can be lost in tumour cell lines cultured under artificial conditions for years, and therefore this discovery of the universality of aerotaxis would not have been possible by studying cancer cell lines alone. Altogether, our results support a putative role for aerotaxis in tumour spread leading to metastasis which is the main obstacle to breast cancer therapies. Hence, we advocate for the use of EGFR inhibitors at the pre-metastatic stage of breast cancer subtypes with the worst prognosis instead of its use at the metastatic stage. These results pave the way for the aerotactic testing of other tumour types such as kidney, non-small cell lung cancer, colorectal cancer in which EGFR plays a prominent role, and in primary metastasis cells in which EGFR activity is impaired.

## Electronic supplementary material

Below is the link to the electronic supplementary material.

Supplementary Material 1 Video 1: Aerotaxis of untransformed MCF10A cells:
This video shows the aerotactic migration of non-transformed MCF10A cells confined under a glass coverslip responsible for hypoxia generation for 48h. For this aerotactic assay, four thousand cells were plated as a 1 μL droplet within a well of a 96-well plate, and following adhesion, the cell cluster was confined under a 6 mm glass coverslip. The video is compiled from an Incucyte 48 h time-lapse experiment using a 4X bright-field objective. Cells located at the border of the cluster migrate directionally towards the edge of the coverslip following the steep oxygen gradient generated by cell respiration while the late cells migrated more randomly since they navigated outside of the oxygen gradient.

Supplementary Material 2 Video 2: Aerotaxis of primary T5 tumour cells.
Epithelial cancer cells were extracted from the T5 tumour and fibroblasts and immune cells were removed by magnetic immunopurification on EpCAM+ MACS columns (Myltenii Biotech, Germany). Aerotaxis of cancer cells from this representative tumour was observed for 48h. As can be seen, the cells at the margin of the cell cluster undergo efficient aerotactic migration in a medium supplemented with EGF while the central cluster in the most hypoxic region, but outside the gradient of oxygen, ends up breaking up into different cell clusters.

Supplementary Material 3 Video 3: Aerotaxis of primary T7 tumour cells.
Another example of aerotaxis of cancer cells extracted from another tumour, the T7 tumour. The migration oberved for 48h is very similar to that of video 2.

Supplementary Material 4 Video 4: Absence of aerotaxis of MCF10A in Cultrex:
Compared to the conditions used in Video 1, Cultrex-BME (extracellular matrix secreted by Engelbreth-Holm-Swarm mouse sarcoma cells polymerizing at 37°C) instead of the conventional liquid medium was used before confining the cell cluster under the glass coverslip. Cells’ migration was observed for 60 h. In contrast to Video 1, we see that MCF10A cells fail to perform aerotactic migration when embedded in Cultrex-BME. The reason is that these untransformed cells are not invasive (see Table 3).

Supplementary Material 5 Video 5: Aerotaxis of primary T6 tumour cells in Cultrex:
The conditions are identical to those of Video 4, Cultrex-BME being used instead of the usual liquid medium for the migration step which was observed for 60 h. Unlike MCF10A cells, primary cancer cells from the T6 tumour embedded in Cultrex-BME can invade the extracellular matrix (ECM) upon cell confinement, demonstrating that aerotaxis is a strong enough signal to guide cancer cells through ECM. Note the star-like trajectory of cells in the ECM instead of the usual ring-shaped migration seen in videos 2 and 3.

Supplementary Material 6 Video 6: Magnification of aerotactic invasion of primary T6 tumour cells in Cultrex:
This video illustrates how primary cancer cells from the T6 tumour may escape from hypoxia when embedded in Cultrex-BME. Leader cells, probably endowed with high matrix degradation skills, open the way within the extracellular matrix to facilitate invasion of additional cells migrating as cohorts.

Supplementary Material 7

## Data Availability

Not applicable.

## Abbreviations

ECM: Extracellular matrix
EGFR: Epithelial Growth Factor receptor
EPO: Erythropoietin
GSH: reduced glutathione
H_2_O_2_: Hydrogen peroxide
HIF: Hypoxia-Inducible Factor
NAC: N-Acetyl Cysteine
OXPHOS: Oxidative Phosphorylation system
PBS: Phosphate-Buffered Saline
PHD: Prolyl-Hydoxylase Domain
ROS: Reactive Oxygen Species
TNBC: Triple Negative Breast Cancer
VEGF: Vascular Endothelial Growth Factor
VHL: von Hippel-Lindau
