# Supplementary material for: EGFR-dependent aerotaxis is a common trait of breast tumour cells
Source: J Exp Clin Cancer Res. 2022 Nov 16;41:324. doi: 10.1186/s13046-022-02514-y (PMC9667613; doi:10.1186/s13046-022-02514-y)
Supplement: Supplementary file 7 — Supplementary Material 7 [file 13046_2022_2514_MOESM7_ESM.pdf]

# Supplementary information, figures and videos

**<u>Sup Figure 1:</u> Migration of T9, T10, T15 and T16 breast tumour cells in the aerotactic assay.**

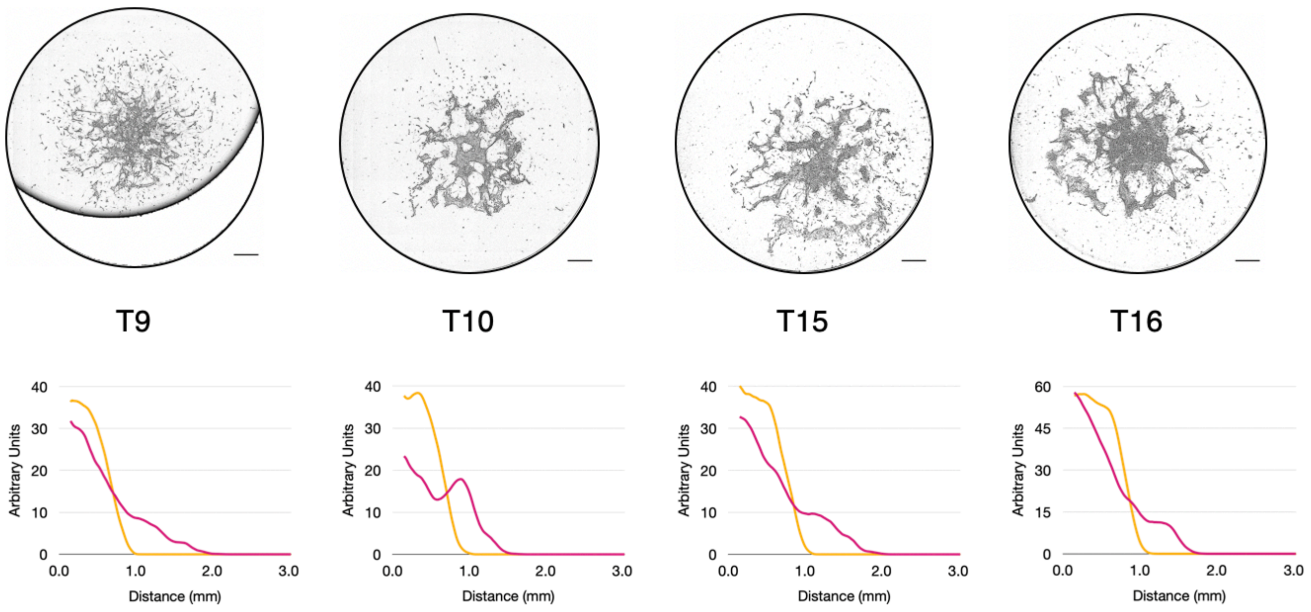


Representative images taken at 48 h corresponding to the aerotactic migration of T9, T10, T15 and T16 tumour cells subjected to the aerotactic assay. Cell distribution from the centre of the well at 0 h (yellow) and 48 h (carmine) are indicated as graphs (mean of three experiments). Y-axis scale is given in arbitrary units. Scale bars, 0.5 mm.

**<u>Sup Figure 2:</u> Comparison of aerotaxis versus intrinsic migration of T5, T6, T8 T10, T12 and T16 breast cancer cells.**

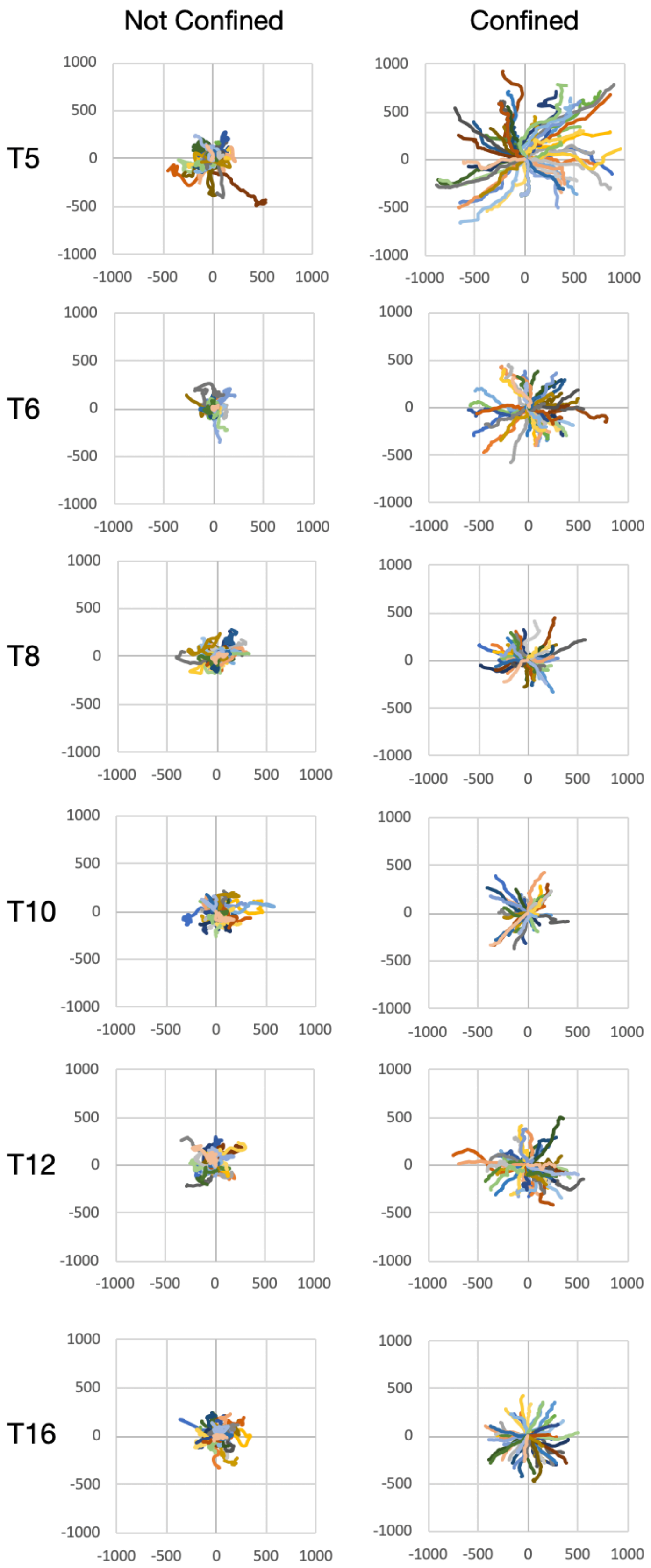


24 h trajectories of 50 representative cells per condition followed as XY plots (scales in µm) of the indicated cells either plated at 5% confluency (not confined) or subjected to the aerotactic assay (confined).

<u>Sup Figure 3:</u> **Migration of breast cancer cells in the aerotactic assay relies on a functional oxidative phosphorylation (OXPHOS) system.**

**a) Tumours T4 to T19:**

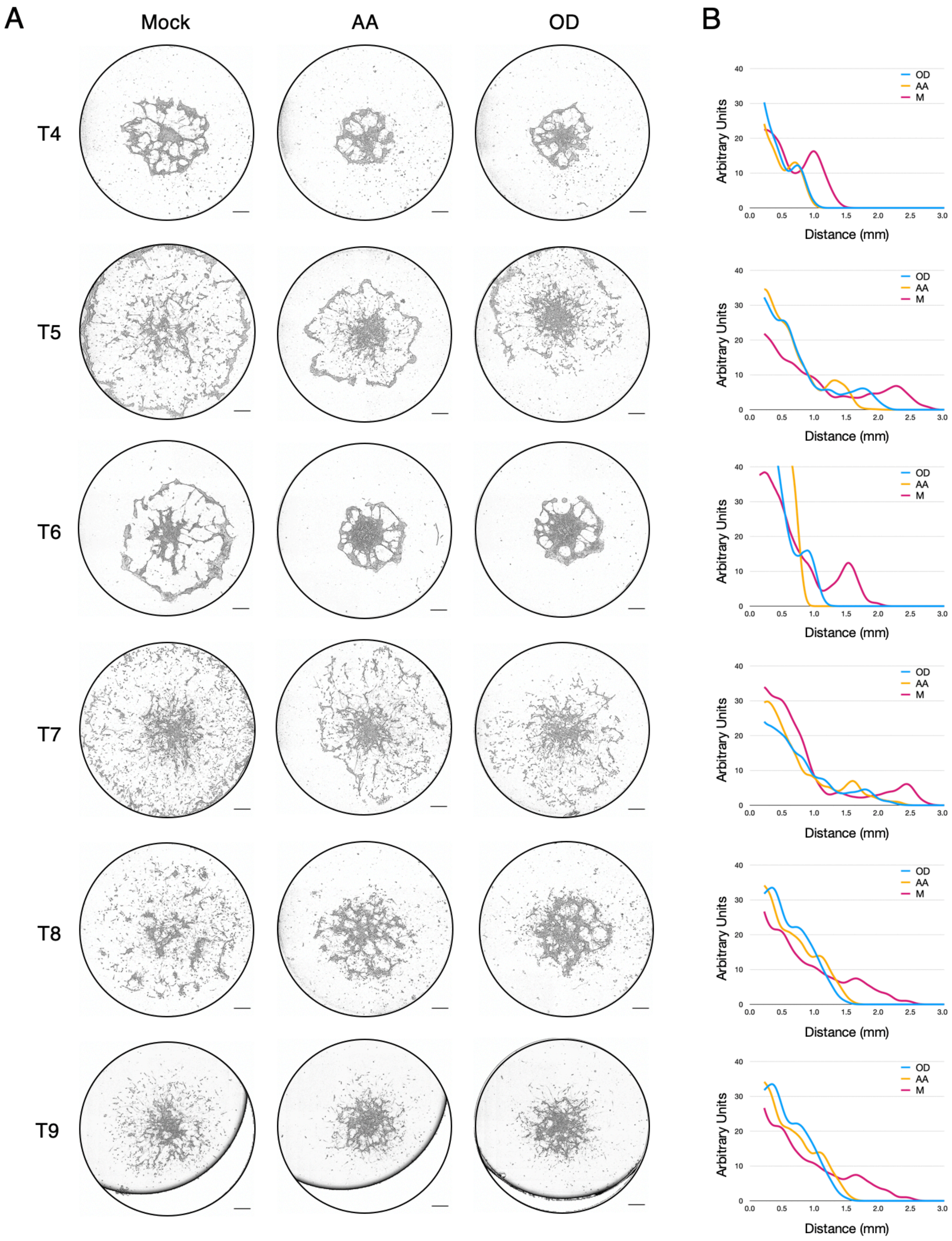

**b) Tumours T10 to T17:**

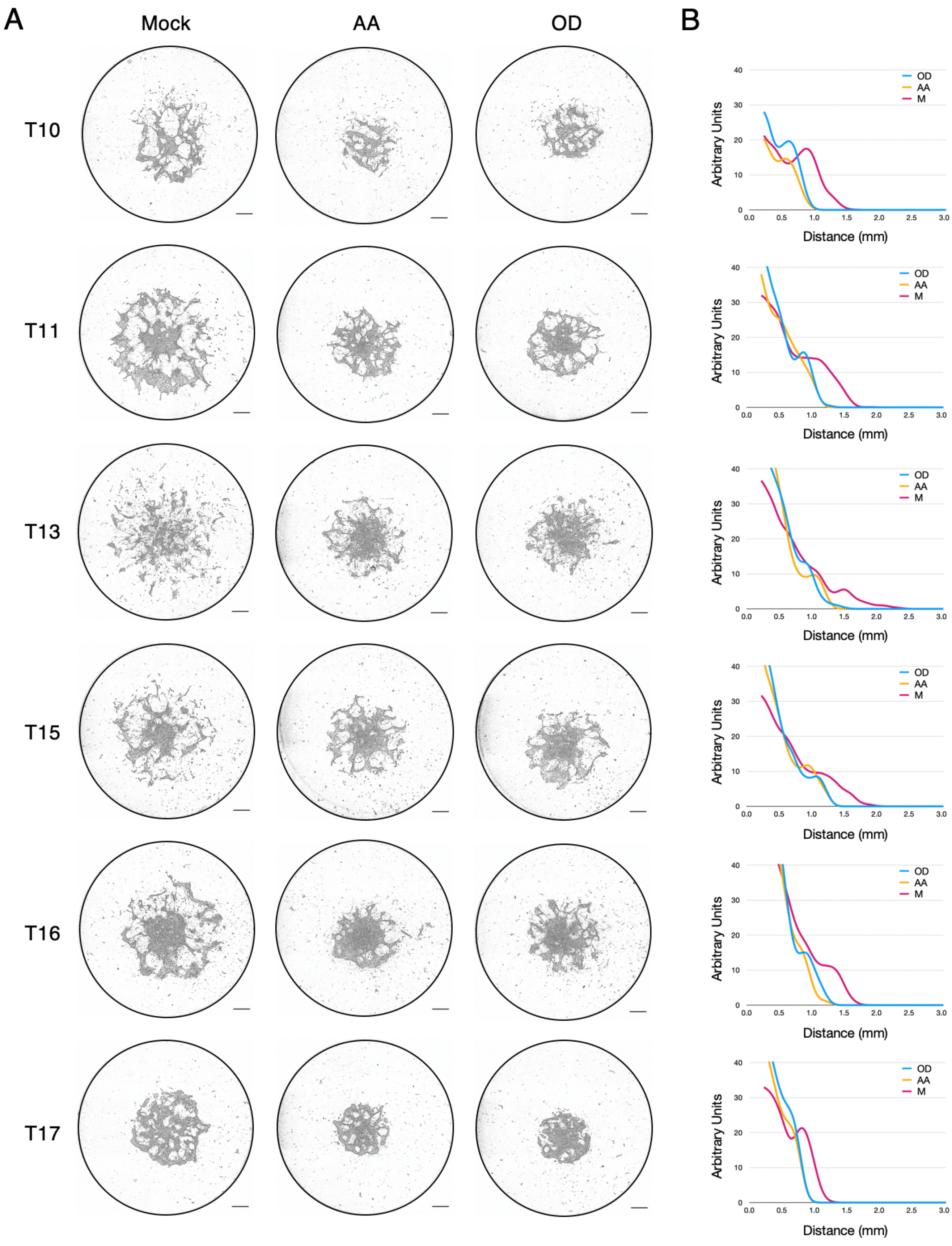


**(A)** Aerotactic migration at 48 h of the indicated tumour cells either untreated (Mock) or treated with Antimycin A (AA, 1 μM) or Oligomycin D (OD, 0.5 μM). Scale bars 0.5 mm. **(B)** Cell distribution from the centre of the well at 48 h in the Mock condition (carmine, mean of three independent experiments) in cells treated with 1 μM AA (yellow) or 0.5 μM OD (blue). Y-axis scale is given in arbitrary units.

**<u>Sup Figure 4:</u> Aerotactic migration is impaired by antioxidants.**

**a) Tumours T4 to T9:**

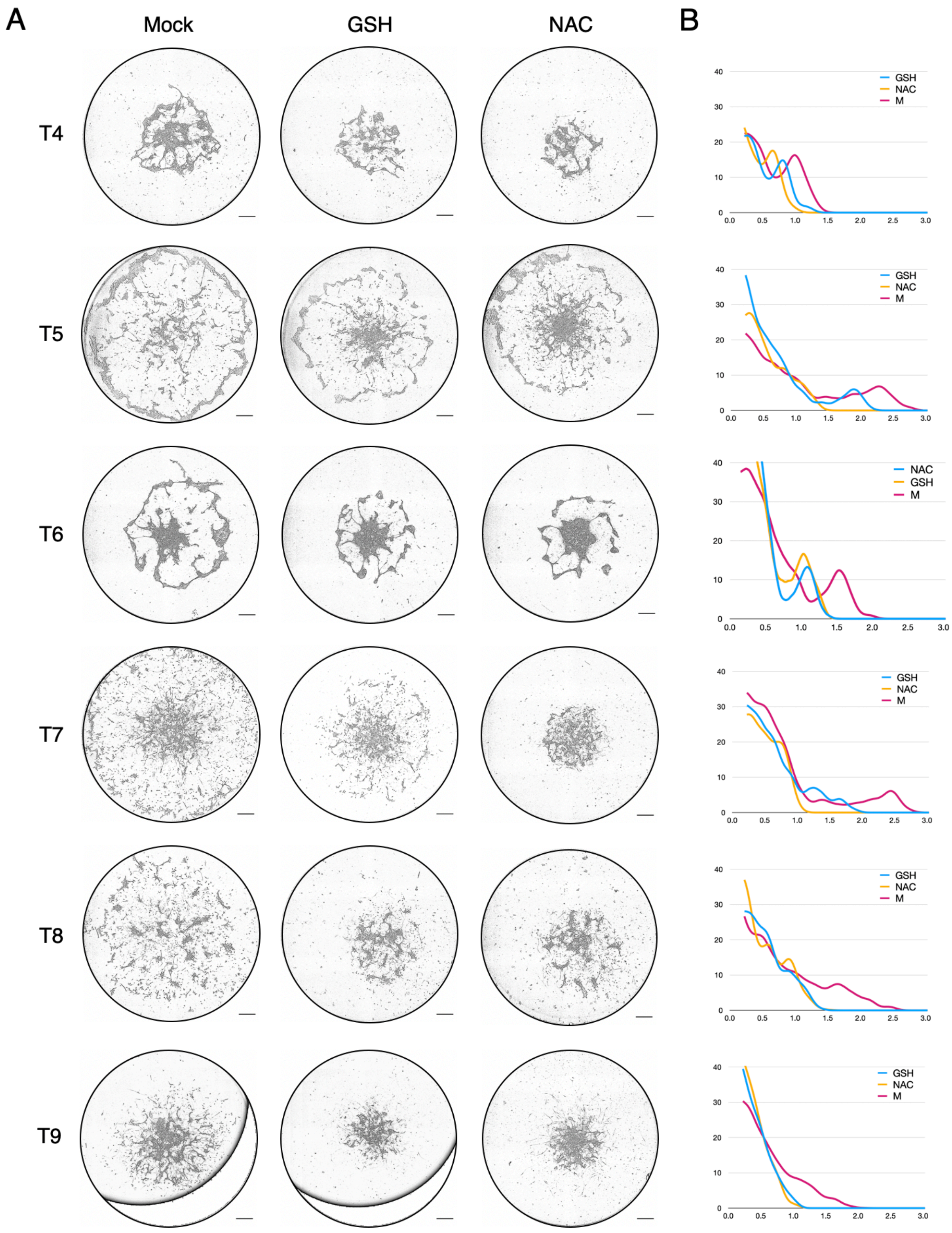

## b) Tumours T10 to T17:

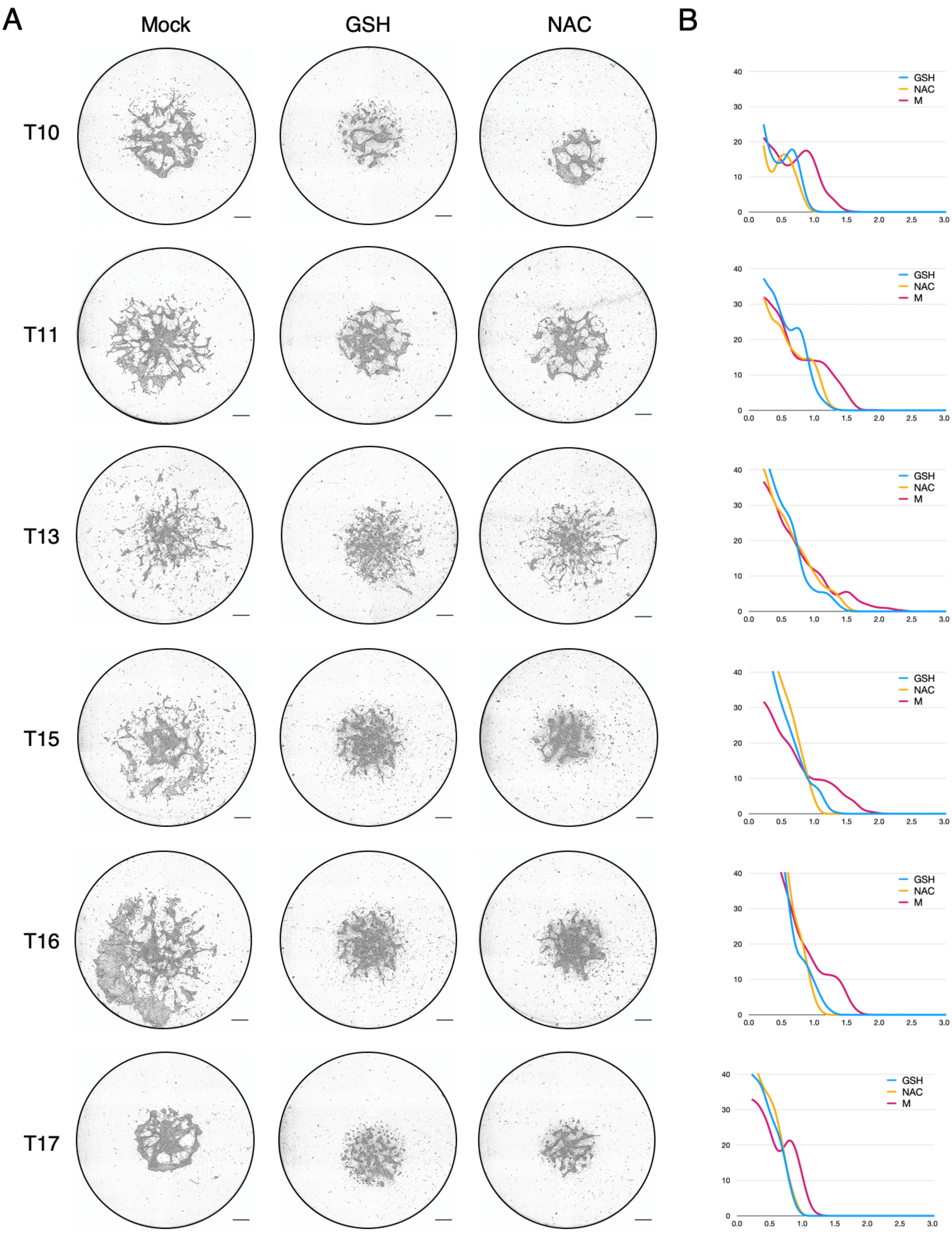


**(A)** Aerotactic migration at 48 h of the indicated tumour cells either untreated (Mock) or treated with 10 mM reduced glutathione (GSH, except for T7 treated with 5 mM GSH) or 10 mM N-Acetyl Cysteine (NAC). Scale bars 0.5 mm. **(B)** Cell distribution from the centre of the cell cluster (in mm) at 48 h in the Mock condition (carmine, mean of three experiments) in cells treated with 10 mM GSH (blue) or 10 mM NAC (yellow). Y-axis scale is given in arbitrary units.

**<u>Sup Figure 5:</u> Re-localization of EGFR following Cetuximab treatment.**

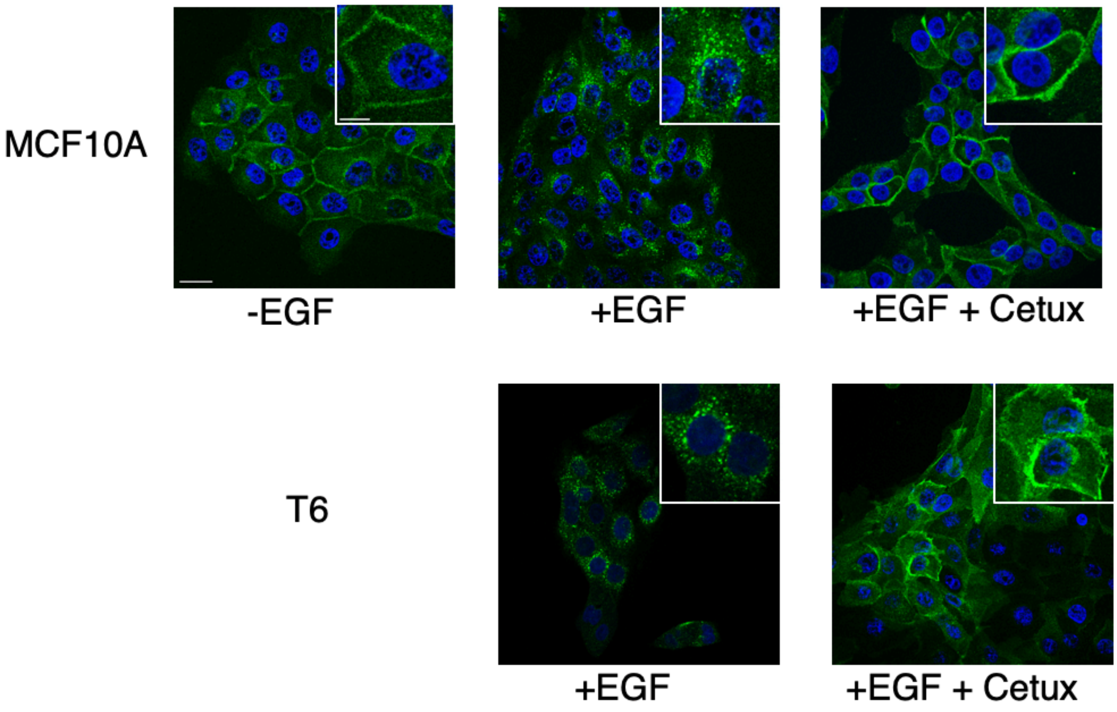


Immunofluorescence imaging of EGFR in the absence of EGF (-EGF), upon activation by EGF (+EGF), or incubated with EGF and Cetuximab, a monoclonal EGFR neutralising antibody (+EGF +Cetux) in MCF10A cells and T6 cells. Scale bars 25 µm and 10 µm for higher magnification views inserted at the upper-right corner of each image.

Sup Figure 6: **Aerotaxis of breast cancer cells is dependent on EGFR activation.**

**a) Tumours T4 to T9:**

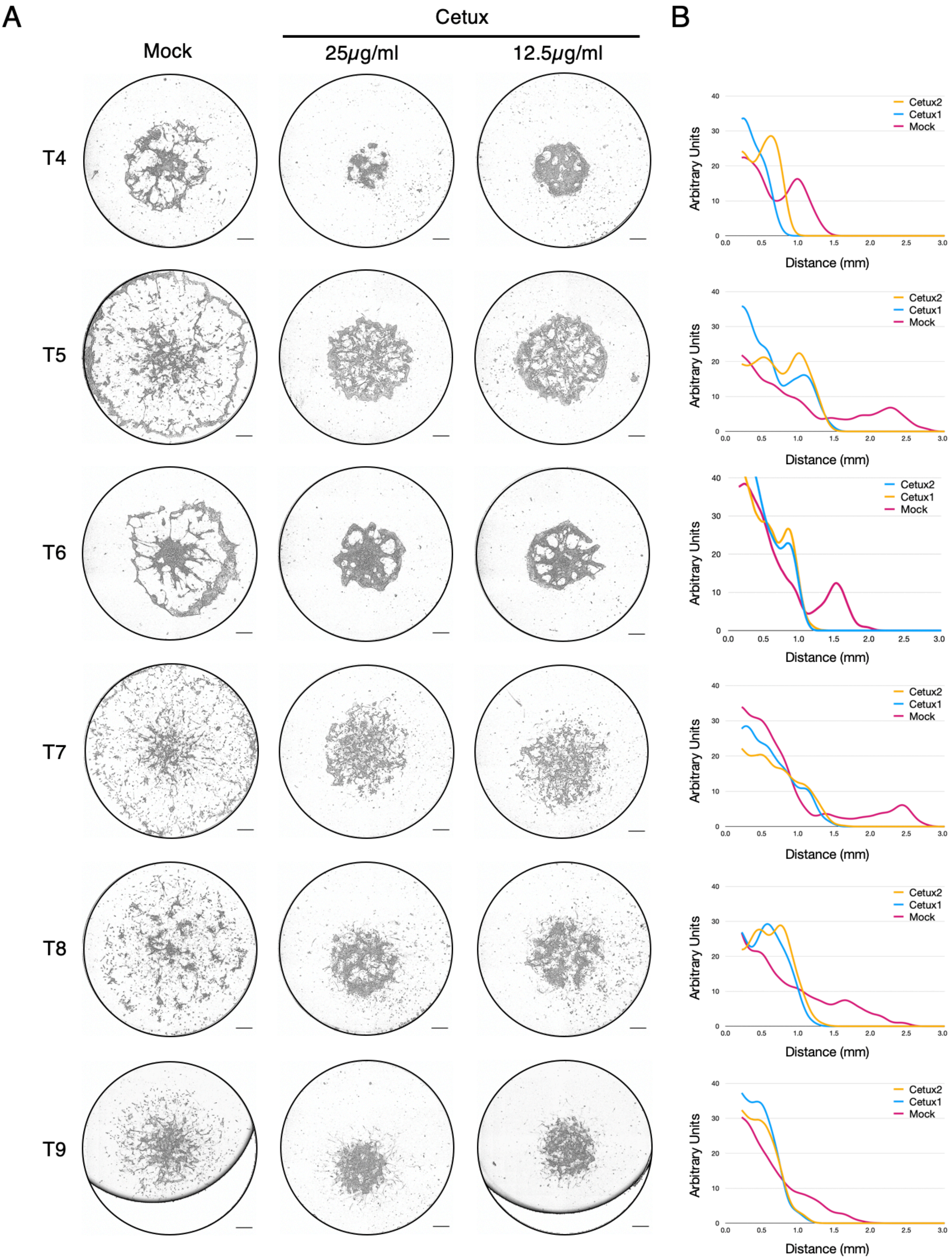

## b) Tumours T10 to T17:

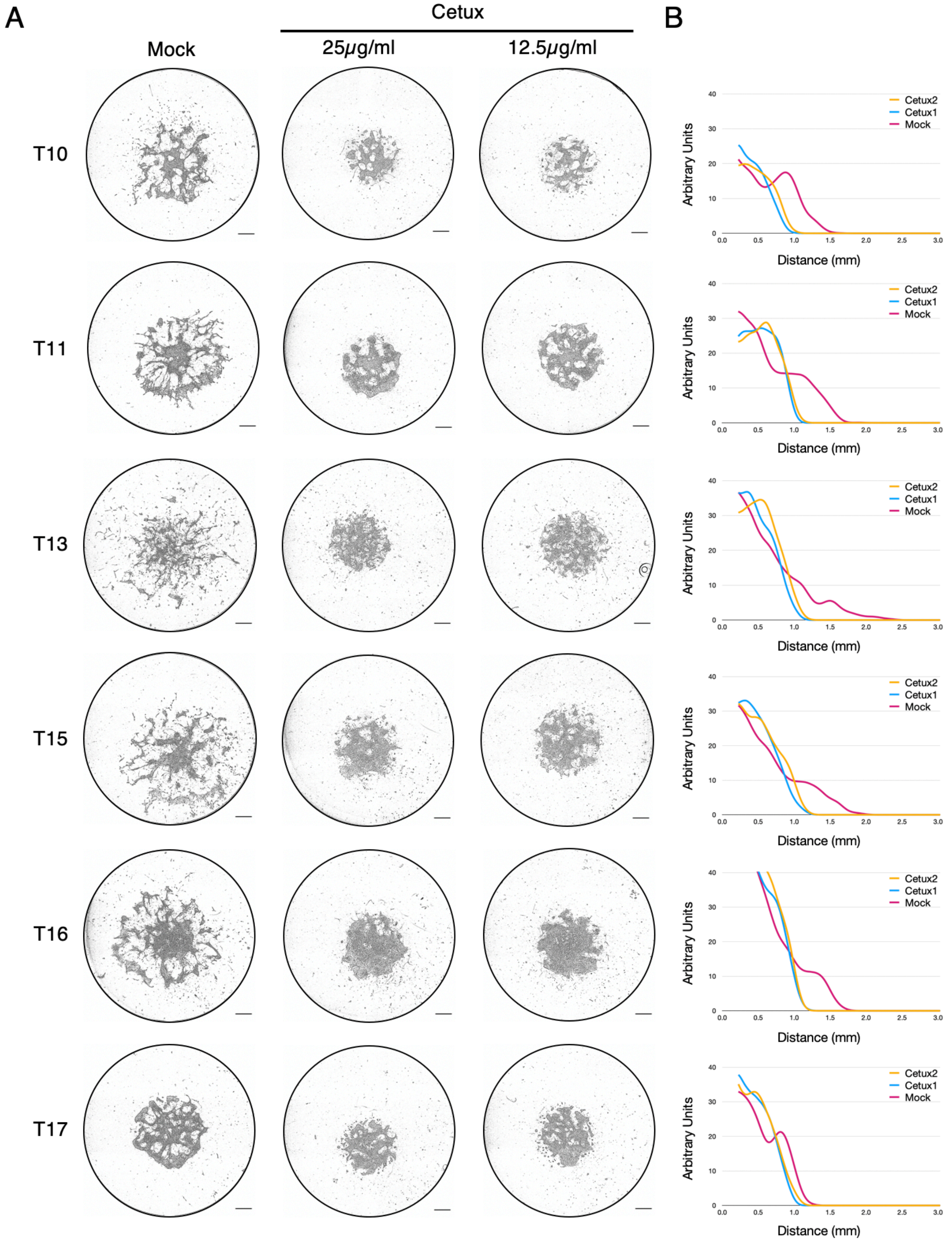


**(A)** Aerotactic migration at 48 h of the indicated tumour cells either untreated (Mock), or treated with 25 µg/mL or 12.5 µg/mL of Cetuximab (Cetux). Scale bars 0.5 mm. **(B)** Cell distribution from the centre of the cell cluster (in mm) at 48 h in the Mock condition (carmine, mean of three experiments) in cells treated with 12.5 µg/ml of cetuximab (yellow) or with 25 µg/ml of cetuximab (blue). Y-axis scale is given in arbitrary units.

**Sup Figure 7: Migration of breast cancer cell lines in the aerotactic assay.**

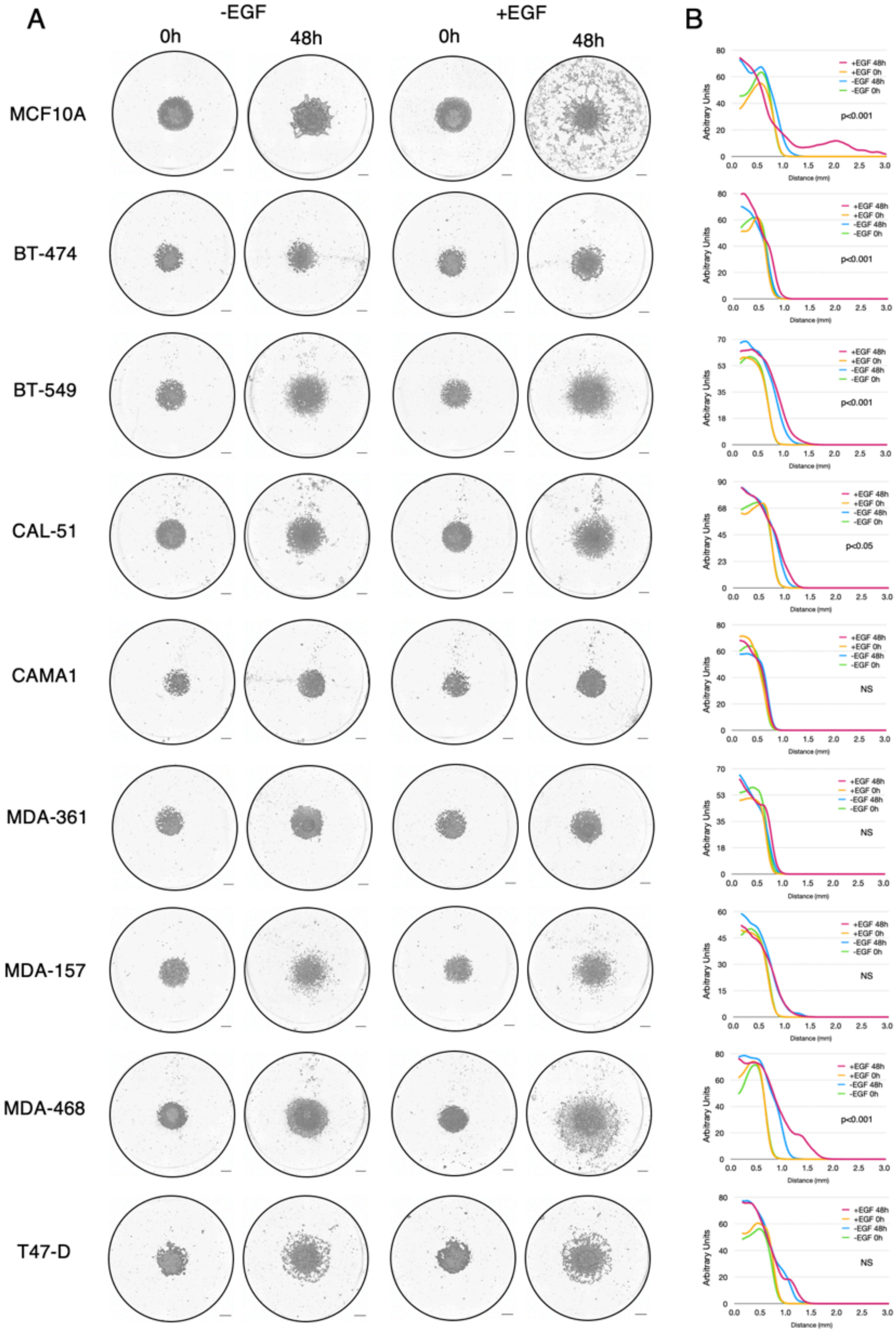

(**A**) Representative images taken at 48 h of breast cancer cell lines subjected to the aerotactic assay in medium minus EGF (-EGF) or supplemented with 10 µg/mL of EGF (+EGF). Scale bars, 0.5 mm. (**B**) Cell distribution from the centre of the cell cluster (in mm) at 0 h (green) and 48 h (carmine) in the -EGF condition and at 0 h (yellow) and 48 h (blue) in the +EGF condition are indicated as graphs (mean of three experiments). Y-axis scale is given in arbitrary units. To assess the difference between the plus and minus EGF conditions at 48 h, a Student t-test statistical was performed on D5% values. P-values are indicated within graphs. NS not significant.

## Video titles and captions:

Video 1: Aerotaxis of untransformed MCF10A cells:
This video shows the aerotactic migration of non-transformed MCF10A cells confined under a glass coverslip responsible for hypoxia generation for 48h. For this aerotactic assay, four thousand cells were plated as a 1 µL droplet within a well of a 96-well plate, and following adhesion, the cell cluster was confined under a 6 mm glass coverslip. The video is compiled from an Incucyte 48 h time-lapse experiment using a 4X bright-field objective. Cells located at the border of the cluster migrate directionally towards the edge of the coverslip following the steep oxygen gradient generated by cell respiration while the late cells migrated more randomly since they navigated outside of the oxygen gradient.

Video 2: Aerotaxis of primary T5 tumour cells.
Epithelial cancer cells were extracted from the T5 tumour and fibroblasts and immune cells were removed by magnetic immunopurification on EpCAM+ MACS columns (Myltenii Biotech, Germany). Aerotaxis of cancer cells from this representative tumour was observed for 48h. As can be seen, the cells at the margin of the cell cluster undergo efficient aerotactic migration in a medium supplemented with EGF while the central cluster in the most hypoxic region, but outside the gradient of oxygen, ends up breaking up into different cell clusters.

Video 3: Aerotaxis of primary T7 tumour cells.
Another example of aerotaxis of cancer cells extracted from another tumour, the T7 tumour. The migration oberved for 48h is very similar to that of video 2.

Video 4: Absence of aerotaxis of MCF10A in Cultrex:
Compared to the conditions used in Video 1, Cultrex-BME (extracellular matrix secreted by Engelbreth-Holm-Swarm mouse sarcoma cells polymerizing at 37°C) instead of the conventional liquid medium was used before confining the cell cluster under the glass coverslip. Cells' migration was observed for 60 h. In contrast to Video 1, we see that MCF10A cells fail to perform aerotactic migration when embedded in Cultrex-BME. The reason is that these untransformed cells are not invasive (see Table 3).

Video 5: Aerotaxis of primary T6 tumour cells in Cultrex:
The conditions are identical to those of Video 4, Cultrex-BME being used instead of the usual liquid medium for the migration step which was observed for 60 h. Unlike MCF10A cells, primary cancer cells from the T6 tumour embedded in Cultrex-BME can invade the extracellular matrix (ECM) upon cell confinement, demonstrating that aerotaxis is a strong enough signal to guide cancer cells through ECM. Note the star-like trajectory of cells in the ECM instead of the usual ring-shaped migration seen in videos 2 and 3.

Video 6: Magnification of aerotactic invasion of primary T6 tumour cells in Cultrex:
This video illustrates how primary cancer cells from the T6 tumour may escape from hypoxia when embedded in Cultrex-BME. Leader cells, probably endowed with high matrix degradation skills, open the way within the extracellular matrix to facilitate invasion of additional cells migrating as cohorts.
